# Natural Molecules and Neuroprotection: Kynurenic Acid, Pantethine and α-Lipoic Acid

**DOI:** 10.3390/ijms22010403

**Published:** 2021-01-02

**Authors:** Fanni Tóth, Edina Katalin Cseh, László Vécsei

**Affiliations:** 1 Department of Neurology, Interdisciplinary Excellence Centre, Faculty of Medicine, University of Szeged, Semmelweis Street 6, H-6725 Szeged, Hungary; toth.fanni@med.u-szeged.hu (F.T.); csehedina.k@gmail.com (E.K.C.); 2 MTA-SZTE, Neuroscience Research Group, Semmelweis Street 6, H-6725 Szeged, Hungary

**Keywords:** neurodegenerative diseases, neuroprotection, kynurenine pathway, kynurenic acid, pantethine, α-lipoic acid, Alzheimer’s disease, Parkinson’s disease, Huntington’s disease, multiple sclerosis

## Abstract

The incidence of neurodegenerative diseases has increased greatly worldwide due to the rise in life expectancy. In spite of notable development in the understanding of these disorders, there has been limited success in the development of neuroprotective agents that can slow the progression of the disease and prevent neuronal death. Some natural products and molecules are very promising neuroprotective agents because of their structural diversity and wide variety of biological activities. In addition to their neuroprotective effect, they are known for their antioxidant, anti-inflammatory and antiapoptotic effects and often serve as a starting point for drug discovery. In this review, the following natural molecules are discussed: firstly, kynurenic acid, the main neuroprotective agent formed via the kynurenine pathway of tryptophan metabolism, as it is known mainly for its role in glutamate excitotoxicity, secondly, the dietary supplement pantethine, that is many sided, well tolerated and safe, and the third molecule, α-lipoic acid is a universal antioxidant. As a conclusion, because of their beneficial properties, these molecules are potential candidates for neuroprotective therapies suitable in managing neurodegenerative diseases.

## 1. Introduction

Neuronal damage in the central nervous system (CNS) is universal in neurodegenerative diseases (NDs) [1]. NDs are defined by the progressive loss of neurons in the CNS, which generates deficits in brain function [2]. The symptoms of NDs vary from memory and cognitive deficits to the deterioration of one’s capability to breath or move [3]. The most frequent NDs are Alzheimer’s disease (AD), Parkinson’s disease (PD), Huntington’s disease (HD), amyotrophic lateral sclerosis (ALS) and multiple sclerosis (MS). The incidence of NDs has increased greatly worldwide due to the rise in life expectancy, and this associates them with profound social and economic burdens [4].

**Figure 1:**
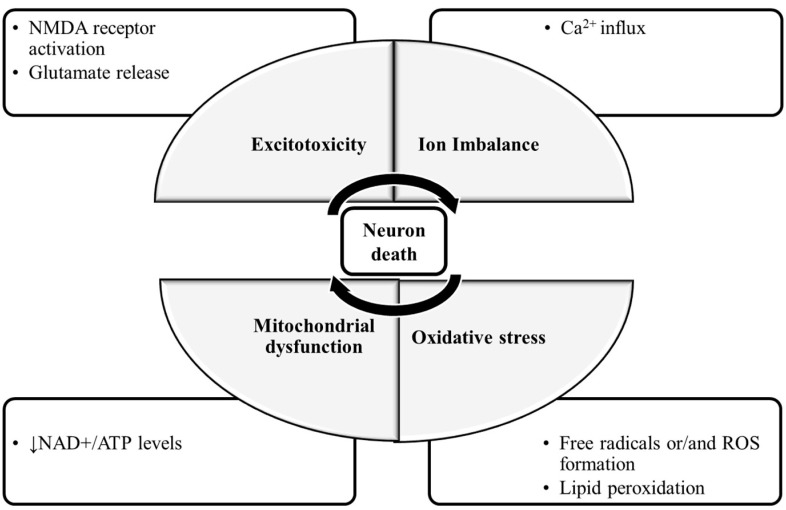
The molecular mechanisms of neuronal damage. The mechanisms of neuronal death are focused on excitatory amino acid release, calcium influx and calcium overload, oxidative stress and mitochondrial dysfunction. During glutamate-induced excitotoxicity, glutamate activates NMDA receptors, leading to a Ca^2+^ influx and overload, which is associated with increased ROS formation and the damaged mitochondria resulting in neuronal death. Abbreviations: NMDA receptor: N-methyl-d-aspartic acid receptor; NAD^+^: nicotinamide adenine dinucleotide; ATP: adenosine triphosphate; ROS: reactive oxygen species.

The molecular mechanisms of neuronal damage are mostly based on excitatory amino acid release and oxidative stress, causing mitochondrial dysfunction [5] (Figure 1). Under physiological conditions, in the CNS, excitatory amino acids are crucial neurotransmitters and their release and uptake are very well controlled. Nonetheless, their accumulation can lead to brain damage [6]. In glutamate-induced excitotoxicity, glutamate activates *N*-methyl-d-aspartic acid receptors (NMDARs), leading to a Ca^2+^ overload [7]. This process is associated with increased reactive oxygen species (ROS), as well as mitochondrial dysfunction resulting in neuronal apoptosis [8,9]. The brain is more vulnerable to damage by oxidative stress due to its high content of polyunsaturated fatty acids [10], that are very prone to ROS attacks [11], which result in lipid peroxidation. Furthermore, the brain has a high rate of O_2_ utilization and a low antioxidant defense, and the accumulated metals like copper or iron are capable of catalyzing the formation of hydroxyl radicals [12].

Neuroprotection denotes approaches that defend the CNS against neuronal injury and/or death while subjected to trauma or neurodegenerative disorders. It slows the progression of the disease and prevents neuronal death [13]. Hence, neuroprotection is a crucial part of care for NDs [14].

Neuroprotection can be classified into three groups: pharmacological-, non-pharmacological- and cellular and genetic approaches. Pharmacological approaches include antioxidants, neurotransmitter agonists/antagonists, anti-inflammatory drugs and natural products [15]. Non-pharmacological approaches include exercise that influences body metabolism [16], diet control to reduce risk factors such as hyperlipidemia [17] and acupuncture, that can help adjust body metabolism and immunity [18]. Cellular and genetic approaches include growth/trophic factors [19]. 

Considering there are various changes that occur in the aging brain, it is implausible that targeting a single change is able to intervene in the complexity of the disease progression. Hence, compounds with multiple biological activities affecting the different age-associated factors that contribute to ND development and progression are extremely needed [20]. The existing therapies available for NDs only relieve symptoms [21]. 

Natural products (including natural molecules) are defined as organic compounds synthesized by living organisms. Some of them are very promising neuroprotective agents because of their structural diversity and wide variety of biological activities [4]. The major neuroprotective targets of natural products and molecules are excitotoxicity, apoptosis, mitochondrial dysfunction, inflammation, oxidative stress and protein misfolding [22,23]. They have anti-neurodegenerative, antioxidant, anti-inflammatory and antiapoptotic effects [24,25]. Natural products and molecules are commonly used as starting points for drug discovery, from which synthetic analogs are synthetized to improve efficacy, potency and to reduce side effects and to increase bioavailability. A lot of the U.S. Food and Drug Administration (FDA)-approved drugs are prompted by natural products [26].

**Figure 2:**
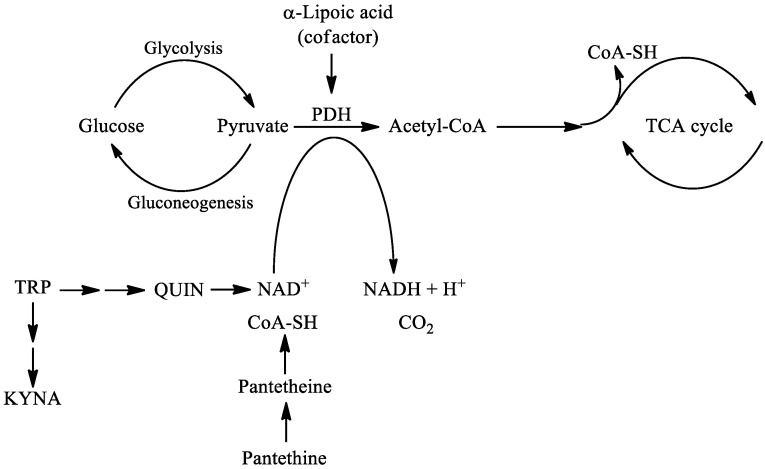
The roles of kynurenine pathway metabolites, pantethine and α-lipoic acid in glycolysis and TCA cycle. PDH is the link between glycolysis and the TCA cycle. α-lipoic acid functions as a cofactor for pyruvate dehydrogenase. The kynurenine pathway of TRP metabolism ultimately leads to the formation of NAD^+^, which will be reduced to NADH. Pantethine is a precursor in the formation of CoA, which functions as an acetyl carrier. It transfers acetyl groups from pyruvate to oxaloacetate, initiating the TCA cycle. Abbreviations: TRP: tryptophan; KYNA: kynurenic acid; QUIN: quinolinic acid; NAD^+^: nicotinamide adenine dinucleotide; CoA: coenzyme A; TCA: tricarboxylic acid; PDH: pyruvate dehydrogenase.

In this review, the following natural molecules are discussed: kynurenic acid, pantethine and α-lipoic acid. In the kynurenine pathway (KP) route of tryptophan (TRP) metabolism, neuroprotective kynurenic acid (KYNA) and the neurotoxic quinolinic acid (QUIN) are formed. QUIN can be further converted to nicotinamide adenine dinucleotide (NAD^+^) [27], which has a cardinal role in energy metabolism [28]. Kynurenines, the metabolites of KP, often have pro- and antioxidant properties with the aromatic hydroxyl acting as an electron acceptor [29]. Pantethine is a neuroprotective reducing agent, it is a precursor in the formation of coenzyme A (CoA). CoA functions as an acetyl carrier. It enables the transfer of acetyl groups from pyruvate to oxaloacetate, initiating the tricarboxylic acid (TCA) cycle [30]. Pyruvate dehydrogenase (PDH) is a mitochondrial matrix multienzyme complex that provides the link between glycolysis and the TCA cycle by catalyzing the conversion of pyruvate to acetyl-CoA [31]. α-lipoic acid (LA) has a redox active disulfide group and in the mitochondria it functions as a cofactor for PDH E2 subunit [32] (Figure 2.). Common features of all three natural molecules include that they are (i) neuroprotective (ii) antioxidant (iii) reducing agents, which have important roles in glycolysis and the TCA cycle. This makes them potential candidates for neuroprotective therapies for managing NDs.

## 2. Kynurenine Pathway, with Focus on Kynurenic Acid

Kynurenines are considered a hot topic nowadays, as in the last 20 years (2000–2020) more than 4600 articles have been published on the topic [33]. 

TRP is an essential amino acid, a building block for protein synthesis and also a precursor for the synthesis of serotonin, KYNA and NAD^+^. The main metabolic route of TRP degradation is through the KP. More than 95% of TRP is metabolized through this route, and only 5% is degraded through the methoxy-indole pathway [34]. The KP is activated by free radicals, interferons and cytokines, which induce the activity of indoleamine 2,3-dioxygenase (IDO) [35] and tryptophan 2,3-dioxygenase (TDO) enzymes [36], the rate-limiting enzymes of the pathway [37].

**Figure 3:**
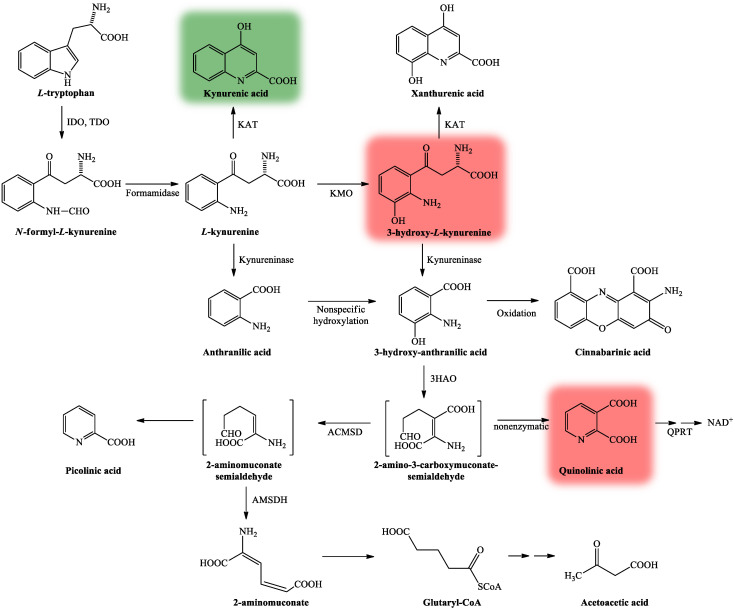
The kynurenine pathway. More than 95% of TRP is metabolized through the KP [34]. The L-tryptophan converting IDO and TDO depict the rate-limiting enzymes of the pathway [37]. KP’s center metabolite is *L*-kynurenine [38] which can be further degraded through three distinct routes to form several neuroactive metabolites: kynurenic acid, 3-hydroxy-*L*-kynurenine and quinolinic acid, which are cardinal in the CNS. Kynurenic acid is formed from *L*-kynurenine in astrocytes and neurons by KATs [39]. Quinolinic acid and 3-hydroxy-*L*-kynurenine are synthesized by infiltrating macrophages and microglia [27]. Neurotoxicity is mediated by quinolinic acid and 3-hydroxy-*L*-kynurenine (color red) via NMDA receptor agonism and free radical production [40], while neuroprotection can be exerted by kynurenic acid (color green) by acting as an antagonist at the NMDA receptor [41]. Abbreviations: TRP: tryptophan; KP: kynurenine pathway; CNS: central nervous system; NMDA receptor: N-methyl-d-aspartic acid receptor; 3HAO: 3-hydroxyanthranilate oxidase; ACMSD: 2-amino-3-carboxymuconate-semialdehyde decarboxylase; AMSDH: 2-aminomuconate-6-semialdehyde dehydrogenase; IDO/TDO: indoleamine 2,3-dioxygenase/tryptophan 2,3-dioxygenase; KAT: kynurenine aminotransferase; KMO: kynurenine 3-monooxygenase; NAD^+^: nicotinamide adenine dinucleotide; QPRT: quinolinic acid phosphoribosyltransferase.

The KP is comprised of several enzymatic steps (Figure 3), which ultimately lead to the formation of NAD^+^, which has a pivotal role in different cellular functions (energy metabolism, gene expression, cell death and regulation of calcium homeostasis) [28]. KP’s center compound is *L*-kynurenine (KYN) [38], which can be further degraded through three different routes, resulting in several neuroactive metabolites. KYNA, the main neuroprotective agent is formed after KYN is catalyzed by the enzyme kynurenine aminotransferase (KAT) [39], whereas QUIN [27] and 3-hydroxy-*L*-kynurenine (3HK) [40] both show neurotoxic properties.

In the CNS, KYNA acts on multiple receptors. KYNA is an endogenous competitive antagonist with high affinity at the strychnine-insensitive glycine-binding NR1 site of NMDARs, it exerts antidepressant and psychotomimetic effects [41,42]. KYNA can bind to the NMDA recognition NR2 site of the receptor as well, albeit with a weaker affinity [43,44]. It also acts upon α-amino-3-hydroxy-5-methyl-4-isoxazole-propionic acid (AMPA) receptors via two distinct mechanisms: at low (nanomolar to micromolar) concentrations, it facilitates AMPA receptor responses, whereas at high (millimolar) concentrations, it competitively antagonizes glutamate receptors [45,46]. KYNA exerts an endogenous agonistic effect on the orphan G protein-coupled receptor (GPR35) [47]. Additionally, KYNA is an endogenous agonist at the aryl hydrocarbon receptor (AHR), expressed in immune cells and in tumor cells [48,49] (Table 1). In 2001, it was suggested by Hilmas et al. that KYNA is a noncompetitive inhibitor of the α7 nicotinic acetylcholine receptor (α-7nAChR) [50]; however, this hypothesis is much debated. The current standpoint is that it does not directly affect nicotinic receptors and results concerning it should only be explained by KYNA’s confirmed sites of action [51].

Since KYNA acts upon multiple receptors, an abnormal decrease or increase in its level may disrupt the equilibrium of neurotransmitter systems, as it can be seen in various neurodegenerative- and neuropsychiatric disorders. KYNA could have therapeutic importance for neurological disorders [52], but since its ability to cross the blood–brain barrier (BBB) is limited [53], its use as a neuroprotective agent is somewhat limited. One option to prevent neurodegenerative diseases includes the influence of the metabolism towards the neuroprotective branch of the KP. Three potential therapeutic strategies for drug development are known: (i) KYNA analogs with better bioavailability and higher affinity to the binding sites of excitatory receptors; (ii) prodrugs of KYNA, which easily cross the BBB combined with an organic acid transport inhibitor to increase brain KYNA levels; and (iii) inhibitors of enzymes of the KP [54].

### 2.1. Kynurenic Acid and Alzheimer’s Disease

AD is a progressive irreversible neurodegenerative disorder, with age-related memory impairments and personality changes. It is known to be the most common cause of dementia [55]. In the pathogenesis of AD, senile plaques which are formed by extracellular deposits of amyloid β peptides (Aβ) and neurofibrillary tangles containing an intracellular assemblage of hyperphosphorylated tau protein play crucial roles [56]. Aβ oligomerization leads to oxidative stress, glutamate excitotoxicity and neuroinflammation, which are all connected to the KP [57]. Alterations in KP leading to a switch towards the production of neurotoxic QUIN have contributed to the pathogenesis of AD. Hence, KP is a potential target for AD therapy [51]. In animal models of AD, the application of 4-Cl-KYN, the BBB-penetrant pro-drug of 7-Cl-KYNA, mitigated hippocampal toxicity caused by QUIN [58]. Another way to enhance the bioavailability of KYNA is to improve its penetration through the BBB [59]. A BBB-permeable KYNA analog was found to be neuroprotective in a *Caenorhabditis elegans* model of AD [60]. Alterations causing elevated KYNA levels in the brain may be associated with cognitive impairments and behavioral alterations [61]. In mice, KAT-II enzyme knock out lead to the improvement of cognitive functions [62].

Regarding human studies, Gulaj et al. [63] found lower TRP and KYNA concentrations and a significant increase in QUIN in AD patient’s plasma compared to controls. 

In AD patients, positive correlations between cognitive function tests and plasma KYNA levels were observed. In contrast, inversely association was found between plasma QUIN levels and cognitive function tests in patients with AD.

In the hippocampus and neocortex of AD patients, high IDO and QUIN expression was found [64]. A correlation between the KYN/TRP ratio and cognitive dysfunction was demonstrated too [65]. In AD patients, lower KYNA levels were found in the serum and erythrocytes, [66] and in the lumbar cerebrospinal fluid (CSF), but no alteration in QUIN levels was found [67] (Table 2).

Aβ1-42 stimulates IDO expression in the human microglia and macrophages where it induces QUIN production [68]. Aβ1-42 and QUIN stimulate cytokine production [69] and increases the hyperphosphorylation of tau proteins via NMDA receptor overactivation, leading to glutamate excitotoxicity in patients with AD [70] and it plays a pivotal role in lipid peroxidation and ROS production, in an NMDAR-dependent or -independent manner, assisting to the pathogenesis of AD [71,72].

Overall, the treatment of AD via the modulation of KP seems to be a logical target of investigation.

### 2.2. Kynurenic Acid and Parkinson’s Disease

PD is a chronic progressive neurodegenerative disorder with a complex dysfunction of the motor network. The pathological characteristic of the disease is the loss of dopaminergic neurons in the substantia nigra pars compacta (SNpc), the development of Lewy bodies [73] and the generation of local inflammation. As inflammatory processes lead to the activation of microglia, the KP is activated, generating QUIN, which leads to the excitotoxic cell death of neurons [74]. Other hallmarks of the pathogenesis of the disorder is mitochondrial dysfunction, which results in oxidative stress and cell energy insufficiency. Immune mechanisms also lead to oxidative stress and apoptosis. Abnormal protein aggregation and glutamate excitotoxicity are also involved in neuronal cell death [75].

Alterations in the KP were seen in animal models of PD. After 1-methyl-4-phenyl-1,2,3,6-tetrahydropyridine (MPTP) treatment, a reduction in the KAT-I activity in the SNpc of mice was observed [76]. The administration of 6-hydroxydopamine (6-OHDA) substantially diminishes KAT-I immunoreactivity in the SNpc neurons [77]. Moreover, 1-methyl-4-phenylpyridinium ion (MPP^+^) caused a decrease in KAT-II activity in rat cerebral cortical slices, leading to the depletion of KYNA [78]. These studies demonstrated the shift of the KP pathway towards 3HK and QUIN, leading to a decline in KYNA levels, generating neurotoxicity and cell death [79]. A pharmacological approach in the treatment of PD could be the modulation of enzymes involved in the KP to increase the level of neuroprotective intermediates and to reduce the neurotoxic ones [80]. KYNA levels are increased by nicotinylalanine, kynureninase and kynurenine 3-monooxygenase (KMO) inhibitor, which protects against NMDA and QUIN toxicity [81]. One approach for increasing KYNA levels in the brain is to prevent the excretion of KYNA from the brain by probenecid [82]. Dopaminergic toxicity was decreased by the co-administration of KYN and probenecid in rats treated with 6-OHDA [83]. Another approach is the use of KYNA analogs or pro-drugs of KYNA. Moreover, 7-Cl-KYNA, a synthetic derivative of KYNA, protected against neurotoxicity caused by QUIN [84]. Glucosamine–kynurenic acid had a similar effect as KYNA, and its effects suggest it might cross the BBB [85]. In cynomolgus monkeys treated with MPTP, a prolonged systemic administration of Ro61-8048, a KMO inhibitor, increased serum KYN and KYNA levels and decreased the development of levodopa (L-dopa)-induced dyskinesias, but it did not influence the anti-Parkinsonian efficacy of L-dopa [86]. 

Regarding the human studies, an altered KP has been found in PD patients [87]. In human post-mortem examinations of PD patients who had taken L-dopa, KYNA levels were significantly reduced in the frontal cortex compared to the control group, whereas KYNA showed lower concentrations in the putamen compared to PD patients who were not treated with L-dopa. In both groups, the KYN levels were reduced in the frontal cortex, in the putamen and in the SNpc compared to controls, this level was found to be the lowest in patients with L-dopa [88]. An increased KYN/TRP ratio was demonstrated in the serum and CSF of PD patients, compared to healthy controls [87]. In the serum of PD patients KAT I and KAT II activities are reduced, with a decreased plasma KYNA level [89] (Table 2). The low level of KYNA has a reduced ability to limit excitotoxicity, through NMDARs, which are induced by QUIN and/or glutamate excess [90]. In PD patients, elevated 3-OH-KYN concentrations were demonstrated in the frontal cortex, putamen and SNpc [88]. In the therapy of PD, some problems remain unsolved: motor and non-motor issues due to therapy, and the need for neuroprotective therapies [91]. The modulation of the KP could possibly be a successful therapeutic strategy for solving these issues. 

### 2.3. Kynurenic Acid and Huntington’s Disease

HD is an autosomal dominantly inherited neurodegenerative disorder with motor, cognitive, and psychiatric symptoms [92]. It is caused by a mutation in the gene coding for the huntingtin protein [93]. The mutant huntingtin protein can sensitize the NMDA receptors [94] and QUIN can act upon the NMDARs and exert damaging effects [95]. Glutamate-induced excitotoxicity plays a crucial role in Huntington’s disease development [96], which can be influenced by kynurenines [97].

In animal models of HD, alterations were observed in the KP. Genetic inhibition of TDO and KMO leads to a neuroprotective shift toward KYNA synthesis and ameliorates neurodegeneration in a *Drosophila melanogaster* model of HD [98]. In a study by Mazarei et al., the inhibition of IDO-1 is likely neuroprotective in HD [99]. A novel KYNA amide compound, which was synthesized in collaboration with our group, *N*-(2-*N*,*N*-dimethylaminoethyl)-4-oxo-1H-quinoline-2-carboxamide hydrochloride, had protective effects in the N171-82Q transgenic HD mouse model where it increased survival, mitigated their hypolocomotion, stopped weight loss and prevented striatal neurons atrophy [100]. At the neuroprotective dose, the KYNA analog did not show any significant side effects, it did not modify the working memory performance, or the long-lasting, consolidated reference memory as opposed to the side effects seen following KYN administration [101,102].

In human studies, IDO-1 activity and KYN levels are increased in the blood of HD patients [103], whereas in their striatum KYNA levels and KAT activity are reduced [104,105]. KYNA levels show a significant decrease in the CSF and in cortex [106,107] (Table 2), whereas 3HK and QUIN levels are increased in the brain of HD patients [108]. The inhibition of KMO ameliorates neurodegeneration in the mice model of HD [109].

Overall, the KP has therefore become an obvious therapeutic target for the treatment of HD [27].

### 2.4. Kynurenic Acid and Amyotrophic Lateral Sclerosis

ALS is a fatal, neurodegenerative disorder that affects the human motor system. Neuroinflammation is important in ALS, causing the death of motor neurons, and it is also associated with excitotoxicity [110], ROS generation, oxidative stress and lipid peroxidation, the latter one being an important feature in QUIN toxicity [111]. 

In ALS patients, significantly increased levels of CSF TRP, KYN and QUIN and decreased levels of serum picolinic acid (PIC) were found [54,112]. In ALS patients with bulbar onset, the CSF KYNA levels were higher compared to controls and patients with limb onset, also in patients with severe clinical status, the CSF KYNA concentrations were elevated compared to the controls, demonstrating KYNA’s neuroprotective role against excitotoxicity [113] (Table 2). At present, the drugs approved for ALS treatment are riluzole, that targets glutamate-mediated excitotoxicity, increasing life expectancy by 2–3 months [114] and edaravone, which is effective in halting ALS progression during the early stages [115]. In identifying candidate drugs for ALS treatment, agents targeting the KP may yield a novel treatment strategy [116].

### 2.5. Kynurenic Acid and Multiple Sclerosis

MS is a progressive, inflammatory, demyelinating disease of the CNS that causes a chronic neurological disability. According to Raine et al. [117], autoreactive T cells and macrophages infiltrate the CNS and attack oligodendrocytes, which myelinate axons, to be the trigger for acute MS lesions. The experimental autoimmune encephalomyelitis (EAE) model is histologically similar to human MS. In the EAE model, numerous disturbances in the KP have been described, i.e., in EAE-induced mice IDO-1 inhibition upon disease induction significantly worsened the disease severity [118], in EAE induced rats increased activity of KMO was observed, whereas KMO inhibition by Ro61-8048 decreased QUIN levels in the spinal cord [119].

In MS patients there is an imbalance in neuroactive and neurotoxic KP metabolites in MS disease pathogenesis [120]. A significant reduction in TRP levels in serum and CSF was observed, indicating the activation of KP in MS [121]. Furthermore, KP activation is a result of cytokines interferon-γ (IFN-γ) and tumor necrosis factor (TNF)-α production, causing IDO-1 expression [122]. In MS patients’ red blood cells, the KAT I and II activities were significantly higher compared to the controls [120]. This rise was correlated with an increase in plasma KYNA levels, which suggests a counterbalancing protective mechanism against neurotoxicity [120]. In the CSF of MS patients during acute relapse, increased KYNA levels were found [123]. In contrast, during the inactive chronic phase a decrease in KYNA concentration was seen [124] (Table 2). Lim et al. [125] have created a predictive model for the disease subtypes using six predictors and metabolomic analysis to profile the KP from the serum of MS patients. The model analyzes the levels of KYNA, QUIN, TRP, PIC, fibroblast growth-factor, and TNF-α to project the disease course with an 85–91% sensitivity. In the future metabolic profiling of the KP may possibly predict clinical course and disease severity [126].

**Table 2:** Kynurenic acid and kynurenine aminotransferase alterations in neurological diseases.

**Alzheimer’s disease**	
Plasma	KYNA ↓ [63]
Serum, erythrocytes	KYNA ↓ [66]
CSF	KYNA ↓ [67]
**Parkinson’s disease**	
Frontal cortex, putamen (L-dopa treatment)	KYNA ↓ [88]
Plasma	KYNA ↓ [89]
Serum	KAT I, KAT II ↓ [89]
**Huntington’s disease**	
Cortex	KYNA ↓ [107]
Striatum	KYNA ↓, KAT ↓ [104,105]
CSF	KYNA ↓ [106]
**Amyotrophic lateral sclerosis**	
CSF (patients with bulbar onset or severe clinical status)	KYNA ↑ [113]
**Multiple sclerosis**	
Plasma	KYNA ↑ [120]
Erythrocytes	KAT I, KAT II ↑ [120]
CSF (patients with acute relapse)	KYNA ↑ [123]
CSF (patients with chronic remission)	KYNA ↓ [124]

Abbreviations: ↓: decrease in level; ↑: increase in level; KYNA: kynurenic acid; CSF: cerebrospinal fluid; KAT: kynurenine aminotransferase.

**Figure 4:**
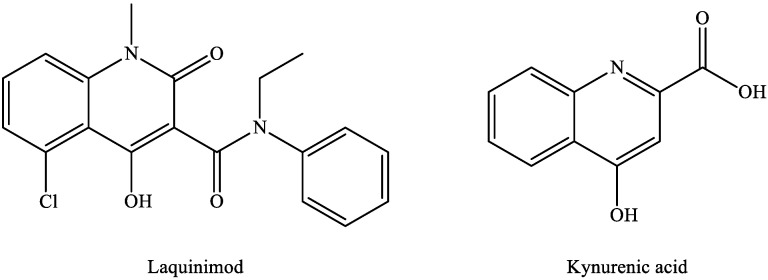
Structural similarities between laquinimod and kynurenic acid.

The available MS treatments are all anti-inflammatory, but there is a need for neuroprotective agents or for drugs that facilitate remyelination. In the search for novel therapeutic candidates by modulating the KP pathway, laquinimod is of particular interest. Laquinimod [127,128] displays a remarkable structural similarity to KYNA (Figure 4), and it ameliorated inflammatory demyelination, metabolic oligodendrocyte injury, with further anti-inflammatory effects in the cuprizone-treated mice, a model of MS [129]. 

It has further shown immunomodulatory and neuroprotective features, rather than immunosuppressive effects in relapsing-remitting MS (RR-MS) patients [130]. Laquinimod was not approved by the Committee for Medicinal Products for Human Use (CHMP) because of severe adverse effects. As reported by the CHMP, in animal studies, a higher occurrence of malignancy was found after long-term exposure to laquinimod. Even though in clinical trials no treatment-related cancer was detected, the CHMP concluded that after laquinimod treatment, long-term cancer risk could not be ruled out. Additionally, in animal studies, laquinimod has a possible teratogenic effect. Because laquinimod only had a modest effect in clinical trials, the CHMP came to the conclusion that the possible risk of long-term laquinimod treatment outweighs its advantageous effect [128]. 

## 3. Pantethine

A common feature of pantethine and tryptophan metabolism is that they have a metabolite somehow connected to the TCA cycle, i.e., in the KP route of TRP metabolism, the neurotoxic compound QUIN will lead to the formation of NAD^+^, whereas pantethine is responsible for the formation of CoA, important in the delivery of the acetyl-group to the TCA cycle. Pantethine’s importance was revealed in 1949, when a new compound, named *Lactobacillus bulgaricus* factor (LBF) was discovered due to its capability to promote the growth of *Lactobacillus bulgaricus*. LBF was universally distributed in the natural materials [131]. LBF was shown to be a fragment of CoA and in the essential growth factor, mercaptoamine was combined with pantothenic acid (Vitamin B5) as an amide [132]. This substance occurs in two forms: pantetheine and pantethine.

**Figure 5:**
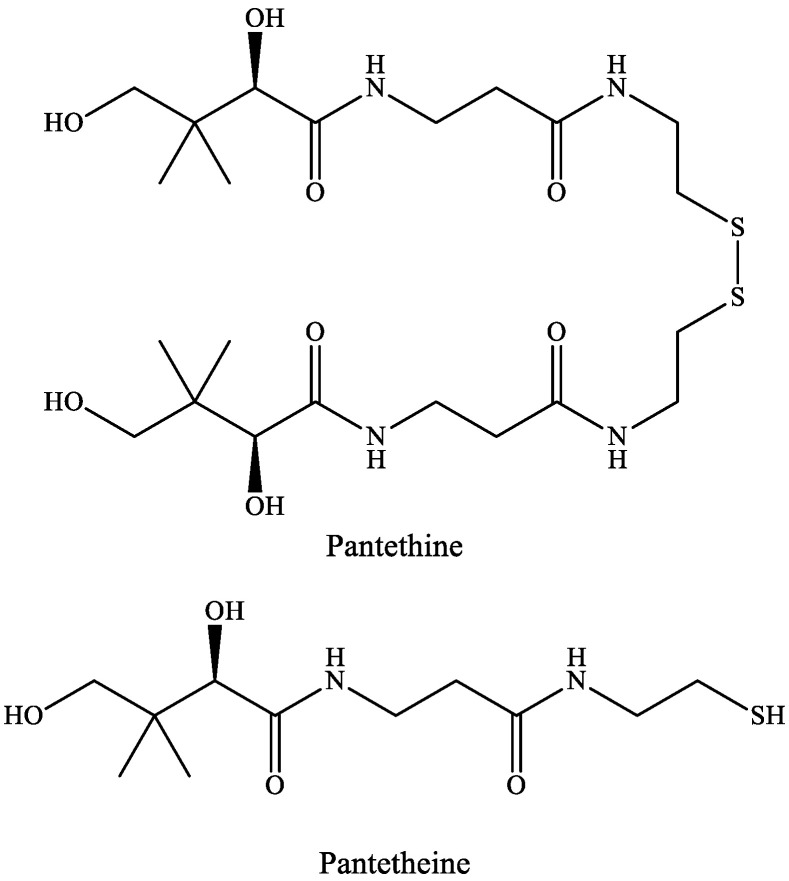
The structure of pantethine and pantetheine.

Pantetheine is the cysteamine amide analog of pantothenic acid (vitamin B_5_) and it is an intermediate in the synthesis of CoA. In pantethine, two molecules of pantetheine are linked by a disulfide bridge. This forms the active part of the CoA molecule (Figure 5). 

Most plants and microorganisms can enzymatically combine pantoic acid with β-alanine to produce pantothenic acid. Mammals are not able to synthesize pantothenic acid since they lack the enzyme. Different foods contain CoA, pantethine, pantetheine and pantothenic acid, so the endogenous synthesis of CoA can begin with pantothenic acid. 

**Figure 6:**
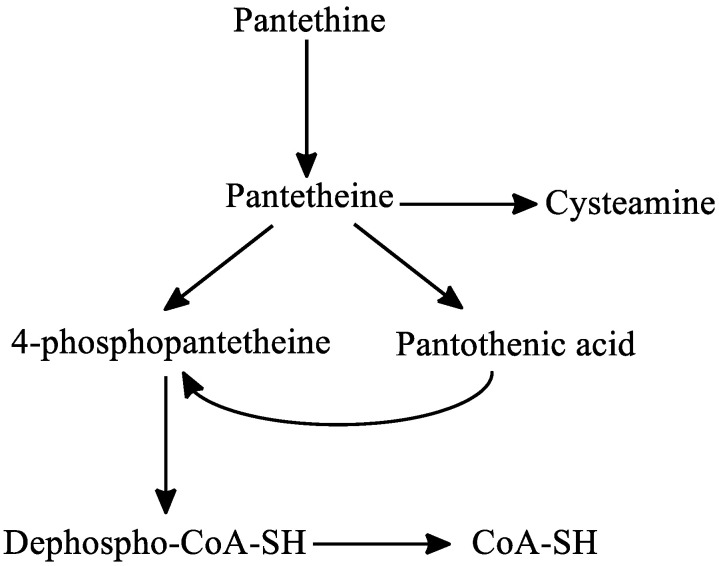
The metabolism of pantethine. Abbreviations: CoA: coenzyme-A.

Regarding the metabolism of pantethine, following oral or intravenous intake, pantethine is immediately hydrolyzed to pantetheine in the small intestine membranes and in blood. Pantetheine can then be phosphorylated to 4′-phosphopantetheine, which is later converted to dephospho-CoA-SH, and finally to CoA-SH in the mitochondria. Pantetheine is transformed to pantothenic acid and cysteamine in hepatocytes. Cysteamine is subsequently metabolized to taurine or is reused to form pantetheine. Pantothenic acid cannot be further degraded in the liver [30,133] (Figure 6).

Since cysteamine in high doses depletes somatostatin and prolactin in different organs, the effect of pantethine was investigated on the level of these hormones in different tissues. Pantethine significantly reduced somatostatin concentrations in the following tissues: duodenal mucosa, gastric mucosa, pancreas, cerebral cortex and hypothalamus [134]. Prolactin was also markedly reduced in the pituitary and in plasma by pantethine, so it can be considered for the management of hyperprolactinemia [135]. In rats, peripherally injected cysteamine and to a lesser extent pantethine reduced noradrenaline and increased dopamine and 3,4-dihydroxyphenylacetic acid (DOPAC) hypothalamic concentrations [136]. Carbon tetrachloride-induced hepatotoxicity in rats is protected by pantethine, pantothenic acid and cystamine. Pantethine provided the greatest protection [137].

### 3.1. Pantethine and Alzheimer’s Disease 

In AD, treatments are mainly based on the reduction in Tau hyperphosphorylation or Aβ. Hence, currently available therapeutic strategies show only moderate symptomatic effects. 

In primary cultured astrocytes of 5XFAD mice, pantethine mitigates metabolic dysfunctions and decreases astrogliosis and IL-1β production [138]. These results associated with pantethine lead to the investigation of its effects in vivo in the 5XFAD (Tg) mouse model of AD. Long-term pantethine treatment significantly reduced glial reactivity and Αβ accumulation and modulated the aggressive attitude of Tg mice. Furthermore, the expression of AD-related genes that are differentially expressed in Tg mice, were significantly mitigated. The expression of a great number of genes involved in the regulation of Αβ processing and synaptic activities, that are downregulated in Tg mice, were recovered after pantethine treatment.

Based on these results, pantethine could be contemplated as a possible therapeutic option for preventing, slowing, or halting AD progression [139].

### 3.2. Pantethine and Parkinson’s Disease

The fact that there is a deficiency in the activity of complex I in the substantia nigra of PD patients establishes the link between PD and mitochondria [140]. In the MPTP-mouse model of PD, pantethine reduced MPTP-induced neurotoxicity in treated mice, by enhancing fatty acid β-oxidation, which causes an increase in the levels of circulating ketone bodies (KB) and an improvement of mitochondrial function [141]. Pantethine protects from MPTP-induced BBB leakage and significantly mitigates clinical scores [142]. 

If TCA cycle activity is low, acetyl-CoA can be used for the biosynthesis of KB via β-hydroxy-methylglutaryl-CoA (HMG-CoA) synthesis. The primary KB are d-β-hydroxybutyrate (dβHB) and acetoacetate (ACA) generated by hepatocytes and they are transported to the tissues, including the brain [142]. *β*-OHB and ACA are protective in a wide range of cerebral injuries and diseases and in vitro they maintain neuronal cell integrity and stability [143]. In another MPTP mouse model, different parameters were studied, such as changes in fatty acid *β*-oxidation, the *L*-3-hydroxybutyryl-CoA dehydrogenase activity and the circulating ketone body (KB) levels, which all showed a decrease, however, these alterations were restored by pantethine treatment with improvement of dopaminergic neuron loss and motility disorders. Pantethine’s protective effect was due to the increase in glutathione (GSH) synthesis, the recovery of mitochondrial complex I activity, adenosine triphosphate (ATP) synthesis and oxygen consumption, resulting in neuroprotection against dopaminergic injury [141]. Pantethine has the same effects as KB administration and ketogenic diets, but with multiple advantages, including the prevention of the damaging effect of the long-term administration of high-fat diets, due to its hypolipidemic properties [144].

This natural compound should also be considered as a potential therapy against PD. 

### 3.3. Pantethine and Major Depressive Disorder 

Major depressive disorder (MDD) is a common psychiatric disorder [145] and it is treated with antidepressants. Unfortunately, current antidepressants in patients have about a 60% response rate [146]. Currently available main classes of antidepressants are tricyclic antidepressants, monoamine oxidase inhibitors, selective serotonin reuptake inhibitors and serotonin-noradrenalin reuptake inhibitors; which all increase the concentration of monoamines in the synaptic cleft [147]. Antidepressants increase central brain-derived neurotrophic factor (BDNF) levels and the activation of the BDNF-signaling pathway might contribute to their therapeutic mechanism [148]. 

The neuroprotective effects of cystamine and cysteamine were previously described [149], but due to serious side-effects, including seizures [150] and hepatic vein thrombosis [151], cysteamine-related agents should be further explored in the treatment of MDD. Among these agents, pantethine may be one of the most promising agents, as it is a naturally occurring substance that can be administered orally with hardly any side effects, and it further metabolizes to cysteamine. Another advantage of pantethine is an anti-arteriosclerotic medicine sold by some pharmaceutical companies [152], and many geriatric depression patients may have an arteriosclerotic etiology [153].

### 3.4. Pantethine and Pantothenate Kinase-Associated Neurodegeneration Syndrome

Pantothenate kinase-associated neurodegeneration (PKAN) syndrome is the most common form of a group of genetic disorders, called neurodegeneration with brain iron accumulation, which are characterized by iron overload in the brain and are diagnosed by radiological and histopathological examinations [154]. PKAN is an autosomal recessive disease, with common features such as dystonia, dysarthria, rigidity, pigmentary retinal degeneration and brain iron accumulation [155]. PKAN is a result of mutations in the PANK2 gene that codes the mitochondrial enzyme pantothenate kinase 2. This enzyme is required for the de novo synthesis of CoA, as it is involved in the phosphorylation of pantothenate [156]. Reduced PANK2 enzymatic activity is proposed to be responsible for the accumulation of cysteine, that can chelate iron, leading to the formation of free radicals [157]. In addition, CoA deficiency and, as a result, defects in phospholipid metabolism may impair the membranes and cause increased oxidative stress, altering iron homeostasis [158]. Unfortunately, to this day the pathophysiology of PKAN is not fully understood, and there is still no cure to halt or reverse the symptoms.

In a PKAN Drosophila model, pantothenate kinase deficiency caused a neurodegenerative phenotype and a reduced lifespan. This Drosophila model revealed that the impairment of pantothenate kinase is linked to decreased levels of CoA, mitochondrial dysfunction and increased protein oxidation. The rescue of the phenotype found in the hypomorph mutant dPANK/fbl is obtained by pantethine feeding, which recovers CoA levels, ameliorates mitochondrial function, rescues brain degeneration, and improves locomotor abilities, and extends lifespan [159]. The zebrafish orthologue of hPANK2 can be found on chromosome 13. The downregulation of pank2 can cause a lack of CoA in zebrafish embryos in specific cells and tissues. Compensation of the wild type phenotype can be obtained by exposing P2-MO-injected embryos to 30 μM pantethine [160]. A Pank2 knockout mouse model did not exactly repeat the human disorder but it showed azoospermia and mitochondrial dysfunctions. This mouse model was challenged with a ketogenic diet to stimulate mitochondrial β-oxidation lipid use. The ketogenic diet could cause a general damage of bioenergetic metabolism in lack of CoA. Only the low glucose and high lipid content diet-fed Pank2 knockout mice developed a PKAN-like syndrome distinguished by significantly altered mitochondria, serious motor dysfunction, neurodegeneration in the CNS and peripheral nervous system. Pank2 knockout mice had structural alteration of muscle morphology, which was similar with that observed in PKAN patients. Pantethine administration was effective in ameliorating the onset of the neuromuscular phenotype observed in Pank2 knockout mice, which were fed a ketogenic diet [155]. 

Overall, these data indicate that pantethine administration to PKAN patients should be contemplated as a potential, safe and non-toxic therapeutic approach.

### 3.5. Other Properties of Pantethine

Pantethine is also effective in alcoholism, hyperlipoproteinemias and dyslipoproteinemias, cystinosis and cataracts.

The oxidation of ethanol yields acetaldehyde, which is involved in the pathogenesis of alcoholic liver disease [161] and alcohol addiction [162]. Pantethine did not cause side effects at clinical doses of 30–600 mg/day. However, it significantly decreased the acetaldehyde levels in blood in healthy (non-flushing) subjects. In contrast, this effect was not found in flushing (alcohol-sensitive) subjects [163]. Chronic ethanol treatment diminished acetyl-CoA availability by inhibiting pantothenic acid incorporation into CoA [164]. In chronic alcoholics there is an association between the diminished Ach level in brain, due to ethanol consumption, and the cognitive and memory impairment. The inhibitory effect of ethanol on brain Ach synthesis can be reversed or prevented by pantothenic acid [165]. Clinical investigations with acute and chronic alcohol intoxicated patients are necessary to clarify the therapeutic effects of pantethine in alcoholism.

There are effective drugs for the management of hyperlipoproteinemias and dyslipoproteinemias available, but their long-term toxicity and hepatopathy may limit their clinical use. Moreover, these diseases require an almost life-long drug administration; so natural products, such as pantethine, have been studied to possibly replace the synthetic drugs. Pantethine has anticatabolic properties and it stimulates fatty acid oxidation. It can normalize dyslipoproteinemia, lower serum lipid levels, and increase the concentration of high-density lipoprotein (HDL) associated cholesterol (Chol) and apo-lipoprotein A-I (Apo A-I) [30]. The total CoA content is increased in perfused rat liver and in liver homogenate by pantethine [166]. The increased availability of CoA leads to an enhancement of the TCA cycle, and so that it stimulates acetate oxidation at the cost of fatty acid and Chol synthesis. Apolipoprotein B (Apo B) is protected against peroxidation in vitro by the sulfhydryl (−SH) containing antioxidative pantethine [167], decreasing the atherogenic Chol concentration in blood [168]. Clinical trials demonstrated that the administration of pantethine at doses ranging between 300 and 600 mg twice daily was successful in the management of patients with familial or sporadic hyperlipidemia [168,169]. Pantethine is significantly more effective than diet in lowering the serum lipid content (triglyceride (TG), total Chol and low-density lipoprotein cholesterol), with an increase in the HDL-Chol level [170]. As a conclusion, pantethine can be an effective treatment of patients with total serum Chol levels > 200 mg/dL and/or serum triacylglycerol levels > 150 mg/dL [30].

Cystinosis is an autosomal recessive genetic disorder with an abnormal accumulation of cystine in the lysosomes of the cells, ultimately leading to the intracellular crystal formation. It is caused by a mutation on chromosome 17 in the CTNS gene that codes for cystinosin, the lysosomal membrane-specific transporter for cysteine. There are three types of cystinosis: infantile cystinosis, intermediate cystinosis, and non-nephropathic (also known as ocular) cystinosis [171]. Therapy has been aimed at decreasing intralysosomal cystine accumulation. Cysteamine can be prescribed for the treatment of cystinosis, but it has a bad taste and odor, side-effects and a low therapeutic index. Pantethine depleted cystine as effectively as cysteamine in cystinotic fibroblast cell cultures, so pantethine can be an alternative for cystinosis treatment [172].

A cataract is an opacification of the lens of the eye, which causes impaired vision. Cataracts form when the proteins in the lens of the eye clump together. In the selenite model for cataract, pantethine inhibited lens opacification during cataract formation [173]. 

Overall, pantethine is a many sided and well tolerated therapeutic agent that appears to deserve much more attention than it has recently received. 

## 4. α-Lipoic Acid

**Figure 7:**
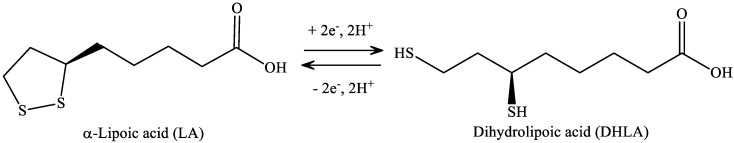
Structure of α-lipoic acid and dihydrolipoic acid.

LA has a redox active disulfide group and it is found naturally in mitochondria as the coenzyme for pyruvate dehydrogenase and α-ketoglutarate dehydrogenase. Small amounts of LA are found in foods (spinach and broccoli) and it is also synthesized in the liver [174]. LA was first isolated in 1951 from bovine liver [175]. Dihydrolipoic acid (DHLA), which is the reduced form of LA, interacts with ROS and reactive nitrogen species (RONS) [176]. Both LA and DHLA have antioxidant effects [177]. LA easily penetrates the BBB [178], after which it is quickly internalized by cells and tissues and is reduced to DHLA [179]. LA is active in aqueous or lipophilic [176] environments. Its conjugate base, lipoate, is more soluble and under physiological conditions it is the most common form of LA. It has a highly negative redox potential of −0.32 V [180], therefore, the redox couple LA/DHLA is a “universal antioxidant” (Figure 7.) [181]. 

LA has antioxidant and anti-inflammatory effects [182]. LA supplementation is effective in animal models in obesity and cardiometabolic disorders. LA produces a decrease in body weight [183]. This effect can be clarified by the suppression of protein kinase 5′ adenosine monophosphate-activated protein kinase (AMPK), its hypothalamic action is pivotal for the regulation of food intake and energy expenditure elevation [184]. In animal models, LA supplementation generated the attenuation of ROS and RONS [185], which are both associated with the reduction in lifespan. The oral supplementation of LA can be a potential supplement in cancer treatment, as it improved survival [186] and reduced unwanted effects of chemotherapy [187]. LA is important in combating inflammation and pain. Positive data on rheumatoid arthritis [188], chronic pain [189], neuropathy [190], migraines [191], ulcerative colitis [192] were published.

In various clinical trials which investigated the therapeutic potential of LA, it was concluded that moderate doses (up to 1800 mg/day) were considered safe. Meanwhile, high doses or intraperitoneally administered LA, at a dosage of 5 to 10 g/day, can elevate hydroperoxide levels in the blood [193].

### 4.1. α-Lipoic Acid and Alzheimer’s Disease

AD’s characteristic features include oxidative stress and energy depletion therefore antioxidants should have positive effects in AD patients. Cultured hippocampal neurons are protected from Aβ-induced neurotoxicity by LA [194], and LA prevents Aβ fibril formation too [195].

In an open clinical study, 600 mg LA was given daily to nine patients with AD (receiving a standard treatment with acetylcholinesterase inhibitors) to examine the influence of LA on the progression of AD. LA treatment stabilized cognitive functions in the patients, shown by constant scores in neuropsychological tests (mini-mental state examination, AD assessment scale and cognitive subscale) [196].

LA has the ability to intervene with pathogenic principles of AD, as it stimulates acetylcholine (ACh) production by activating choline acetyltransferase and elevating glucose uptake, as a result providing more acetyl-CoA for ACh production [197]. LA might represent a potential neuroprotective therapy for AD.

### 4.2. α-Lipoic Acid and Parkinson’s Disease

The activation of microglia, and the accompanying oxidative stress and neuroinflammation play a pivotal role in the pathogenesis of PD [198,199]. In experimental models of PD, lipopolysaccharide (LPS) can be used to activate glial cells [200]. Nasal LPS-induced PD is completely inflammation-driven, and it effectively replicates the chronic, progressive PD pathology [201]. LA can block the LPS-induced inflammatory process [202]. LA administration ameliorated motor dysfunction, preserved dopaminergic neurons and reduced SN α-synuclein accumulation. In M1 microglia, LA blocked nuclear factor-κB activation and the expression of pro-inflammatory molecules. Further neuroprotective action of LA was studied in an experimental model of PD, induced in male Wistar rats by the intrastriatal injection of 6-OHDA. LA improved learning and memory performance and neuromuscular coordination. LA significantly reduced lipid peroxidation levels and recovered the catalase activity and dopamine levels that were damaged by 6-OHDA administration all which lead to a reduction in oxidative stress [203]. It can be concluded that LA displays significant antiparkinsonian effects. 

### 4.3. α-Lipoic Acid and Huntington’s Disease

There is a link between HD pathogenesis and the mitochondrial energetic defect. In HD post-mortem brain sections, respiratory chain deficits were found and this resulted in the use of mitochondrial complex-II (succinate dehydrogenase, SDH) inhibitors to create toxicity models that reproduced HD striatal pathology in vivo [204]. By using toxin 3-nitropropionic acid (3-NP), an irreversible inhibitor of SDH, it was concluded that mitochondrial dysfunctions contribute to the pathogenesis of HD [205]. 3-NP induces neuropathological changes similar to those observed in HD. In the 3-NP rat model of HD, the neuroprotective effect of LA and acetyl-L-carnitine (ALCAR) on 3-NP-induced alterations in the mitochondrial structure, lipid composition, and memory functions was investigated. The combined supplementation of LA + ALCAR improved mitochondrial lipid composition, blocked mitochondrial structural changes, and mitigated cognitive deficits in 3-NP-treated animals. Thus, a combined supplementation of LA + ALCAR can be a possible therapeutic strategy in HD management [206].

Excitotoxicity and oxidative damage are involved in the pathogenesis of HD. LA dietary supplementation increases unbound lipoic acid, its antioxidant effect ameliorates oxidative stress in vitro and in vivo [179]. It was examined whether LA exerted neuroprotective effects in transgenic mouse models of HD. LA generated significant increases in survival in R6/2 and N171-82Q transgenic mouse models of HD. 

These results indicate that LA may have valuable effects in HD patients [207].

### 4.4. α-Lipoic Acid and Multiple Sclerosis

In MS, oxidative stress, and the excess of ROS or RONS are among the contributors to neuronal- and axonal injury [208,209,210]. Animal model studies showed LA treatment decreased matrix metalloproteinase (MMP), IFN-γ, and interleukin-4 (IL-4) [211] and the reduced expression of soluble cell adhesion molecule (ICAM) in spinal cord tissue [212]. Two animal studies showed that LA had dose-dependent effects, with higher dosages (100 μg/mL versus 25 μg/mL) being more effective and oral administration being less effective than injections [211,212].

Studies investigating the effects of LA on demyelination and axonal damage in optic nerve, spinal cord, and brain reported that LA-treated EAE animals had reduced damage in the CNS, which was timing- and route of administration-dependent [211,213,214]. LA administration by intraperitoneal (i.p.) injection seven days or directly after immunization protected axons from demyelination and damage [211,213]. Delayed LA administration also decreased damage to the optic nerve but not as profoundly as the immediate treatment [213]. Oral administration was only protective immediately, but not delayed after EAE immunization [211]. LA treatment led to a reduced disease severity in EAE model animals [215]. 

Concerning the effects of LA on anti-oxidant/inflammatory mediators, a reduction in T cell infiltration into the CNS was found after LA treatment in the spinal cord [214], optic nerve [213], and cerebellum [216]. In terms of the effects of LA on mediators of inflammation, like MMP-9 or ICAM, in MS patients they observed varying results. In the serum of MS patients, 1200 mg LA administered daily for 14 days did not alter the serum levels of MMP-9, a tissue inhibitor of metalloproteinases (TIMP-1), or ICAM [217]. In contrast, another group after 12 weeks of AL treatment, observed marked reductions in IFN-γ, ICAM-1, TGF-β, and IL [218]. 

The use of LA is desirable in numerous areas of health. LA has various beneficial effects on the aging process and neurological disorders. More evaluation is needed to better instruct health professionals on the safety of prescribing LA as a supplement.

## 5. Conclusions

In conclusion, the extensive research and development of natural products including KYNA, pantethine and α-lipoic acid, will lead to information which will potentially enable novel drug discovery. The neuroprotective property of these compounds makes them worthy of much more attention and contemplates them as a possible therapeutic option for several neurological diseases.

## Figures and Tables

**Table 1:** Main binding sites of kynurenic acid.

Receptor	Action
AHR	Agonist
GPR35	Agonist
NMDAR (glycine-2 co agonist NR1 site)	Antagonist
NMDAR (glutamate/NMDA NR2 site)	Antagonist
AMPAR	Agonist/antagonist (dose-dependent)

Abbreviations: GPR35: G protein-coupled receptor 35; AHR: aryl hydrocarbon receptor; NMDAR: *N*-methyl-d-aspartic acid receptor; AMPAR: α-amino-3-hydroxy-5-methyl-4-isoxazolepropionic acid receptor.

## Abbreviations

Aβ	amyloid β peptides
ACA	acetoacetate
ACh	acetylcholine
ACMSD	2-amino-3-carboxymuconate-semialdehyde decarboxylase
AD	Alzheimer’s disease
AHR	aryl hydrocarbon receptor
ALCAR	acetyl-L-carnitine
α-7nAChR	α7 nicotinic acetylcholine receptor
ALS	amyotrophic lateral sclerosis
AMPA	α-amino-3-hydroxy-5-methyl-4-isoxazole-propionic acid
AMPK	5′ adenosine monophosphate-activated protein kinase
AMSDH	2-aminomuconate-6-semialdehyde dehydrogenase
Apo A-I	apo-lipoprotein A-I
Apo B	apolipoprotein B
ATP	adenosine triphosphate
BBB	blood–brain barrier
BDNF	brain-derived neurotrophic factor
CHMP	Committee for Medicinal Products for Human Use
Chol	cholesterol
CNS	central nervous system
CoA	coenzyme A
CSF	cerebrospinal fluid
dβHB	d-β-hydroxybutyrate
DHLA	dihydrolipoic acid
DOPAC	3,4-dihydroxyphenylacetic acid
EAE	experimental autoimmune encephalomyelitis
FDA	U.S. Food and Drug Administration
GPR35	G protein-coupled receptor 35
GSH	glutathione
3HAO	3-hydroxyanthranilate 3,4-dioxygenase
HD	Huntington’s disease
HDL	high-density lipoprotein
3HK	3-hydroxy-L-kynurenine
HMG-CoA	β-hydroxy-methylglutaryl-CoA
ICAM	soluble cell adhesion molecule
IDO	indoleamine 2,3-dioxygenase
IFN-γ	interferon-γ
IL-4	interleukin-4
i.p. injection	intraperitoneal injection
KAT	kynurenine aminotransferase
KB	ketone body
KGDHC	α-ketoglutarate dehydrogenase complex
KMO	kynurenine 3-monooxygenase
KP	kynurenine pathway
KYN	L-kynurenine
KYNA	kynurenic acid
LA	α-lipoic acid
LBF	*Lactobacillus bulgaricus* Factor
L-dopa	levodopa
LPS	lipopolysaccharide
MDD	major depressive disorder
MMP-9	matrix metalloproteinase-9
MPP^+^	1-methyl-4-phenylpyridinium ion
MPTP	1-methyl-4-phenyl-1,2,3,6-tetrahydropyridine
MS	multiple sclerosis
NAD^+^	nicotinamide adenine dinucleotide
ND	neurodegenerative diseases
NMDARs	*N*-methyl-d-aspartic acid receptors
3-NP	3-nitropropionic acid
6-OHDA	6-hydroxydopamine
PD	Parkinson’s disease
PDH	pyruvate dehydrogenase
PIC	picolinic acid
PKAN	pantothenate kinase-associated neurodegeneration
QPRT	quinolinic acid phosphoribosyltransferase
QUIN	quinolinic acid
RONS	reactive oxygen and nitrogen species
ROS	reactive oxygen species
RR MS	relapsing-remitting MS
SDH	succinate dehydrogenase
-SH	sulfhydryl
SNpc	substantia nigra pars compacta
TCA	tricarboxylic acid cycle
TDO	tryptophan 2,3-dioxygenase
TG	triglyceride
TGF-β	tumor growth factor-β
TIMP1	tissue inhibitor of metalloproteinases
TNF-α	tumor necrosis factor-α
TRP	tryptophan

## References

[1] Fei F., Su N., Li X., Fei Z. (2020). Neuroprotection mediated by natural products and their chemical derivatives. Neural Regen. Res..

[2] Angeloni C., Vauzour D. (2019). Natural products and neuroprotection. Int. J. Mol. Sci..

[3] Erkkinen M.G., Kim M.-O., Geschwind M.D. (2018). Clinical neurology and epidemiology of the major neurodegenerative diseases. Cold Spring Harb. Perspect. Biol..

[4] González-Cofrade L., de las Heras B., Ticona L.A., Palomino O.M. (2019). Molecular targets involved in the neuroprotection mediated by terpenoids. Planta Med..

[5] Mattiasson G., Shamloo M., Gido G., Mathi K., Tomasevic G., Yi S., Warden C.H., Castilho R.F., Melcher T., Gonzalez-Zulueta M. (2003). Uncoupling protein-2 prevents neuronal death and diminishes brain dysfunction after stroke and brain trauma. Nat. Med..

[6] Rama R., García J.C., Schaller B. (2016). Excitotoxicity and Oxidative Stress in Acute Stroke. Ischemic Stroke.

[7] Choi D.W. (1994). Calcium and excitotoxic neuronal injury. Ann. N. Y. Acad. Sci..

[8] Lau A., Tymianski M. (2010). Glutamate receptors, neurotoxicity and neurodegeneration. Pflügers Arch. Eur. J. Physiol..

[9] Yildiz-Unal A., Korulu S., Karabay A. (2015). Neuroprotective strategies against calpain-mediated neurodegeneration. Neuropsychiatr. Dis. Treat..

[10] Rice-Evans C., Burdon R. (1993). Free radical-lipid interactions and their pathological consequences. Prog. Lipid Res..

[11] Barrera G. (2012). Oxidative stress and lipid peroxidation products in cancer progression and therapy. ISRN Oncol..

[12] Sakr H.F., Abbas A.M., El Samanoudy A.Z. (2015). Effect of vitamin E on cerebral cortical oxidative stress and brain-derived neurotrophic factor gene expression induced by hypoxia and exercise in rats. J. Physiol. Pharmacol..

[13] Cai W., Zhang K., Li P., Zhu L., Xu J., Yang B., Hu X., Lu Z., Chen J. (2017). Dysfunction of the neurovascular unit in ischemic stroke and neurodegenerative diseases: An aging effect. Ageing Res. Rev..

[14] Brahmachari G., Brahmachari G. (2018). Discovery and Development of Neuroprotective Agents from Natural Products: An Overview. Discovery and Development of Neuroprotective Agents from Natural Products.

[15] Chang R.C.C., Ho Y.S. (2019). Introductory Chapter: Concept of Neuroprotection—A New Perspective. Neuroprotection.

[16] Liu Y., Yan T., Chu J.M.-T., Chen Y., Dunnett S., Ho Y.-S., Wong G.T.-C., Chang R.C.-C. (2019). The beneficial effects of physical exercise in the brain and related pathophysiological mechanisms in neurodegenerative diseases. Lab. Invest..

[17] Appleton J.P., Scutt P., Sprigg N., Bath P.M. (2017). Hypercholesterolaemia and vascular dementia. Clin. Sci..

[18] Cao Y., Zhang L.-W., Wang J., Du S.-Q., Xiao L.-Y., Tu J.F., Liu C.-Z. (2016). Mechanisms of acupuncture effect on Alzheimer’s disease in animal based researches. Curr. Top. Med. Chem..

[19] Semkova I., Krieglstein J. (1999). Neuroprotection mediated via neurotrophic factors and induction of neurotrophic factors. Brain Res. Rev..

[20] Maher P. (2019). The potential of flavonoids for the treatment of neurodegenerative diseases. Int. J. Mol. Sci..

[21] Di Paolo M., Papi L., Gori F., Turillazzi E. (2019). Natural products in neurodegenerative diseases: A great promise but an ethical challenge. Int. J. Mol. Sci..

[22] Leonoudakis D., Rane A., Angeli S., Lithgow G.J., Andersen J.K., Chinta S.J. (2017). Anti-Inflammatory and neuroprotective role of natural product securinine in activated glial cells: Implications for Parkinson’s disease. Mediat. Inflamm..

[23] Deshpande P., Gogia N., Singh A. (2019). Exploring the efficacy of natural products in alleviating Alzheimer’s disease. Neural Regen. Res..

[24] Angeloni C., Giusti L., Hrelia S. (2019). New neuroprotective perspectives in fighting oxidative stress and improving cellular energy metabolism by oleocanthal. Neural Regen. Res..

[25] Flanagan E., Müller M., Hornberger M., Vauzour D. (2018). Impact of flavonoids on cellular and molecular mechanisms underlying age-related cognitive decline and neurodegeneration. Curr. Nutr. Rep..

[26] Western Oregon University https://wou.edu/chemistry/courses/online-chemistry-textbooks/ch105-consumer-chemistry/ch105-chapter-6-hydrocarbons/.

[27] Bohár Z., Toldi J., Fülöp F., Vécsei L. (2015). Changing the face of kynurenines and neurotoxicity: Therapeutic considerations. Int. J. Mol. Sci..

[28] Ying W. (2006). NAD+ and NADH in cellular functions and cell death. Front. Biosci..

[29] Zhuravlev A.V., Zakharov G.A., Shchegolev B.F., Savvateeva-Popova E.V. (2017). Antioxidant properties of kynurenines: Density functional theory calculations. PLoS Comput. Biol..

[30] Horváth Z., Vécsei L. (2009). Current medical aspects of pantethine. Ideggyogy Szle..

[31] Patel M.S., Roche T.E. (1990). Molecular biology and biochemistry of pyruvate dehydrogenase complexes. FASEB J..

[32] Taylor M.R., Hurley J.B., Van Epps H.A., Brockerhoff S.E. (2004). A zebrafish model for pyruvate dehydrogenase deficiency: Rescue of neurological dysfunction and embryonic lethality using a ketogenic diet. Proc. Natl. Acad. Sci. USA.

[33] Web of Science. https://www.webofknowledge.com.

[34] Vécsei L., Szalárdy L., Fülöp F., Toldi J. (2013). Kynurenines in the CNS: Recent advances and new questions. Nat. Rev. Drug Discov..

[35] Hayaishi O. (1976). Properties and function of indoleamine 2,3-dioxygenase. J. Biochem..

[36] Schröcksnadel K., Wirleitner B., Winkler C., Fuchs D. (2006). Monitoring tryptophan metabolism in chronic immune activation. Clin. Chim. Acta.

[37] Mackay G.M., Forrest C.M., Stoy N., Christofides J., Egerton M., Stone T.W., Darlington L.G. (2006). Tryptophan metabolism and oxidative stress in patients with chronic brain injury. Eur. J. Neurol..

[38] Wirleitner B., Neurauter G., Schröcksnadel K., Frick B., Fuchs D. (2003). Interferon-γ- induced conversion of tryptophan: Immunologic and neuropsychiatric aspects. Curr. Med. Chem..

[39] Guidetti P., Amori L., Sapko M.T., Okuno E., Schwarcz R. (2007). Mitochondrial aspartate aminotransferase: A third kynurenate-producing enzyme in the mammalian brain. J. Neurochem..

[40] Németh H., Toldi J., Vécsei L. (2005). Role of kynurenines in the central and peripherial nervous systems. Curr. Neurovasc. Res..

[41] Parsons C.G., Danysz W., Quack G., Hartmann S., Lorenz B., Wollenburg C., Baran L., Przegalinski E., Kostowski W., Krzascik P. (1997). Novel systemically active antagonists of the glycine site of the N-methyl-D-aspartate receptor: Electrophysiological, biochemical and behavioral characterization. J. Pharmacol. Exp. Ther..

[42] Oxenkrug G.F. (2010). Tryptophan-kynurenine metabolism as a common mediator of genetic and environmental impacts in major depressive disorder: The serotonin hypothesis revisited 40 years later. Isr. J. Psychiatry Relat. Sci..

[43] Kessler M., Terramani T., Lynch G., Baudry M. (1989). A glycine site associated with N-methyl-D-aspartic acid receptors: Characterization and identification of a new class of antagonists. J. Neurochem..

[44] Szalárdy L., Zádori D., Toldi J., Fülöp F., Klivényi P., Vécsei L. (2012). Manipulating kynurenic acid levels in the brain—On the edge between neuroprotection and cognitive dysfunction. Curr. Top. Med. Chem..

[45] Prescott C., Weeks A.M., Staley K.J., Partin K.M. (2006). Kynurenic acid has a dual action on AMPA receptor responses. Neurosci. Lett..

[46] Rózsa E., Robotka H., Vécsei L., Toldi J. (2008). The Janus-face kynurenic acid. J. Neural. Transm..

[47] Wang J., Simonavicius N., Wu X., Swaminath G., Reagan J., Tian H., Ling L. (2006). Kynurenic acid as a ligand for orphan G protein-coupled receptor GPR35. J. Biol. Chem..

[48] DiNatale B.C., Murray I.A., Schroeder J.C., Flaveny C.A., Lahoti T.S., Laurenzana E.M., Omiecinski C.J., Perdew G.H. (2010). Kynurenic acid is a potent endogenous aryl hydrocarbon receptor ligand that synergistically induces interleukin-6 in the presence of inflammatory signaling. Toxicol. Sci..

[49] Denison M.S., Nagy S.R. (2003). Activation of the aryl hydrocarbon receptor by structurally diverse exogenous and endogenous chemicals. Ann. Rev. Pharmacol. Toxicol..

[50] Hilmas C., Pereira E.F., Alkondon M., Rassoulpour A., Schwarcz R., Albuquerque E.X. (2001). The brain metabolite kynurenic acid inhibits alpha7 nicotinic receptor activity and increases non-alpha7 nicotinic receptor expression: Physiopathological implications. J. Neurosci..

[51] Stone T.W. (2020). Does kynurenic acid act on nicotinic receptors? An assessment of the evidence. J. Neurochem..

[52] Stone T.W. (2001). Kynurenines in the CNS: From endogenous obscurity to therapeutic importance. Prog. Neurobiol..

[53] Fukui S., Schwarcz R., Rapoport S.I., Takada Y., Smith Q.R. (1991). Blood-brain barrier transport of kynurenines: Implications for brain synthesis and metabolism. J. Neurochem..

[54] Füvesi J., Rajda C., Bencsik K., Toldi J., Vécsei L. (2012). The role of kynurenines in the pathomechanism of amyotrophic lateral sclerosis and multiple sclerosis: Therapeutic implications. J. Neural. Transm..

[55] Sharma R., Razdan K., Bansal Y., Kuhad A. (2018). Rollercoaster ride of kynurenines: Steering the wheel towards neuroprotection in Alzheimer’s disease. Expert Opin. Ther. Targets.

[56] Robakis N.K., Vlamos P., Alexiou A. (2015). Molecular Neuropathology of Alzheimer Dementia and Therapeutic Approaches. GeNeDis 2014 Neurodegeneration; Advances in Experimental Medicine and Biology.

[57] Kincses Z.T., Toldi J., Vécsei L. (2010). Kynurenines, neurodegeneration and Alzheimer’s disease. J. Cell. Mol. Med..

[58] Wu H.Q., Lee S.C., Schwarcz R. (2000). Systemic administration of 4-chlorokynurenine prevents quinolinate neurotoxicity in the rat hippocampus. Eur. J. Pharmacol..

[59] Robotka H., Németh H., Somlai C., Vécsei L., Toldi J. (2005). Systemically administered glucosamine-kynurenic acid, but not pure kynurenic acid, is effective in decreasing the evoked activity in area CA1 of the rat hippocampus. Eur. J. Pharmacol..

[60] Deora G.S., Kantham S., Chan S., Dighe S.N., Veliyath S.K., McColl G., Parat M.O., McGeary R.P., Ross B.P. (2017). Multifunctional analogs of kynurenic acid for the treatment of Alzheimer’s disease: Synthesis, pharmacology, and molecular modeling studies. ACS Chem. Neurosci..

[61] Vécsei L., Beal M.F. (1991). Comparative behavioral and pharmacological studies with centrally administered kynurenine and kynurenic acid in rats. Eur. J. Pharmacol..

[62] Potter M.C., Elmer G.I., Bergeron R., Albuquerque E.X., Guidetti P., Wu H.Q., Schwarcz R. (2010). Reduction of endogenous kynurenic acid formation enhances extracellular glutamate, hippocampal plasticity, and cognitive behavior. Neuropsychopharmacology.

[63] Gulaj E., Pawlak K., Bien B., Pawlak D. (2010). Kynurenine and its metabolites in Alzheimer’s disease patients. Adv. Med. Sci..

[64] Guillemin G.J., Brew B.J., Noonan C.E., Takikawa O., Cullen K.M. (2005). Indoleamine 2,3-dioxygenase and quinolinic acid immunoreactivity in Alzheimer’s disease hippocampus. Neuropathol. Appl. Neurobiol..

[65] Widner B., Leblhuber F., Walli J., Tilz G.P., Demel U., Fuchs D. (2000). Tryptophan degradation and immune activation in Alzheimer’s disease. J. Neural. Transm..

[66] Hartai Z., Juhász A., Rimanóczy A., Janáky T., Donkó T., Dux L., Penke B., Tóth G.K., Janka Z., Kálmán J. (2007). Decreased serum and red blood cell kynurenic acid levels in Alzheimer’s disease. Neurochem. Int..

[67] Heyes M.P., Saito K., Crowley J.S., Davis L.E., Demitrack M.A., Der M., Dilling L.A., Elia J., Kruesi M.J.P., Lackner A. (1992). Quinolinic acid and kynurenine pathway metabolism in inflammatory and non-inflammatory neurological disease. Brain.

[68] Guillemin G.J., Smythe G.A., Veas L.A., Takikawa O., Brew B.J. (2003). A beta 1–42 induces production of quinolinic acid by human macrophages and microglia. NeuroReport.

[69] Walker D.G., Link J., Lue L.F., Dalsing-Hertnandez J.E., Boyes B.E. (2006). Gene expression changes by amyloid beta peptide-stimulated human postmortem brain microglia identify activation of multiple inflammatory processes. J. Leukoc. Biol..

[70] Rahman A., Ting K., Cullen K.M., Braidy N., Brew B.J., Guillemin G.J. (2009). The excitotoxin quinolinic acid induces tau phosphorylation in human neurons. PLoS ONE.

[71] Montine T.J., Neely M.D., Quinn J.F., Beal M.F., Markesbery W.R., Roberts L.J., Morrow J.D. (2002). Lipid peroxidation in aging brain and Alzheimer’s disease. Free Radic. Biol. Med..

[72] St’astny F., Lisy V., Mares V., Lisa V., Balcar V.J., Santamaria A. (2004). Quinolinic acid induces NMDA receptor-mediated lipid peroxidation in rat brain microvessels. Redox Rep..

[73] Braak H., Del Tredici K., Rüb U., de Vos R.A.I., Steur E.N.J., Braak E. (2003). Staging of brain pathology related to sporadic Parkinson’s disease. Neurobiol. Aging.

[74] Lovelace M.D., Varney B., Sundaram G., Lennon M.J., Lim C.K., Jacobs K., Guillemin G.J., Brew B.J. (2017). Recent evidence for an expanded role of the kynurenine pathway of tryptophan metabolism in neurological diseases. Neuropharmacology.

[75] Kincses Z.T., Vécsei L. (2011). Pharmacological therapy in Parkinson’s disease: Focus on neuroprotection. CNS Neurosci. Ther..

[76] Knyihár-Csillik E., Csillik B., Pákáski M., Krisztin-Péva B., Dobó E., Okuno E., Vécsei L. (2004). Decreased expression of kynurenine aminotransferase-I (KAT-I) in the substantia nigra of mice after 1-methyl-4-phenyl-1,2,3,6-tetrahydropyridine (MPTP) treatment. Neuroscience.

[77] Knyihár-Csillik E., Chadaide Z., Mihály A., Krisztin-Péva B., Fenyő R., Vécsei L. (2006). Effect of 6-hydroxydopamine treatment on kynurenine aminotransferase-I (KAT-I) immunoreactivity of neurons and glial cells in the rat substantia nigra. Acta Neuropathol..

[78] Luchowski P., Luchowska E., Turski W.A., Urbanska E.M. (2002). 1-Methyl-4-phenylpyridinium and 3-nitropropionic acid diminish cortical synthesis of kynurenic acid via interference with kynurenine aminotransferases in rats. Neurosci. Lett..

[79] Lim C.K., Fernández-Gomez F.J., Braidy N., Estrada C., Costa C., Costa S., Bessede A., Fernandez-Villalba E., Zinger A., Herrero M.T. (2017). Involvement of the kynurenine pathway in the pathogenesis of Parkinson’s disease. Prog. Neurobiol..

[80] Szabó N., Kincses Z.T., Toldi J., Vécsei L. (2011). Altered tryptophan metabolism in Parkinson’s disease: A possible novel therapeutic approach. J. Neurol. Sci..

[81] Miranda A.F., Boegman R.J., Beninger R.J., Jhamandas K. (1997). Protection against quinolinic acid-mediated excitotoxicity in nigrostriatal dopaminergic neurons by endogenous kynurenic acid. Neuroscience.

[82] Vámos E., Vörös K., Zádori D., Vécsei L., Klivényi P. (2009). Neuroprotective effects of probenecid in a transgenic animal model of Huntington’s disease. J. Neural Transm..

[83] Silva-Adaya D., Perez-De La Cruz V., Villeda-Hernandez J., Carrillo-Mora P., Gonzalez-Herrera I.G., Garcia E., Colín-Barenque L., Pedraza-Chaverrí J., Santamaría A. (2011). Protective effect of l-kynurenine and probenecid on 6-hydroxydopamine-induced striatal toxicity in rats: Implications of modulating kynurenate as a protective strategy. Neurotoxicology Teratol..

[84] Foster A.C., Willis C.L., Tridgett R. (1990). Protection against N-methyl-D-aspartate receptormediated neuronal degeneration in rat brain by 7-chlorokynurenate and 3-amino-1-hydroxypyrrolid-2-one, antagonists at the allosteric site for glycine. Eur. J. Neurosci..

[85] Füvesi J., Somlai C., Németh H., Varga H., Kis Z., Farkas T., Károly N., Dobszay M., Penke Z., Penke B. (2004). Comparative study on the effects of kynurenic acid and glucosamine-kynurenic acid. Pharmacol. Biochem. Behav..

[86] Gregoire L., Rassoulpour A., Guidetti P., Samadi P., Bedard P.J., Izzo E., Schwarcz R., Di Paolo T. (2008). Prolonged kynurenine 3-hydroxylase inhibition reduces development of levodopa-induced dyskinesias in parkinsonian monkeys. Behav. Brain Res..

[87] Widner B., Leblhuber F., Fuchs D. (2002). Increased neopterin production and tryptophan degradation in advanced Parkinson’s disease. J. Neural Transm..

[88] Ogawa T., Matson W.R., Beal M.F., Myers R.H., Bird E.D., Milbury P., Saso S. (1992). Kynurenine pathway abnormalities in Parkinson’s disease. Neurology.

[89] Hartai Z., Klivényi P., Janáky T., Penke B., Dux L., Vécsei L. (2005). Kynurenine metabolism in plasma and in red blood cells in Parkinson’s disease. J. Neurol. Sci..

[90] Zinger A., Barcia C., Herrero M.T., Guillemin G.J. (2011). The involvement of neuroinflammation and kynurenine pathway in Parkinson’s disease. Parkinson’s Dis..

[91] Rákoczi K., Klivényi P., Vécsei L. (2009). Neuroprotection in Parkinson’s disease and other neurodegenerative disorders: Preclinical and clinical findings. Ideggyogy Szle..

[92] Ghosh R., Tabrizi S.J. (2015). Clinical aspects of Huntington’s disease. Curr. Top. Behav. Neurosci..

[93] The Huntington’s Disease Collaborative Research Group (1993). A novel gene containing a trinucleotide repeat that is expanded and unstable on Huntington’s disease chromosomes. Cell.

[94] Chen N., Luo T., Wellington C., Metzler M., McCutcheon K., Hayden M.R., Raymond L.A. (1999). Subtype-specific enhancement of NMDA receptor currents by mutant huntingtin. J. Neurochem..

[95] de Carvalho L.P., Bochet P., Rossier J. (1996). The endogenous agonist quinolinic acid and the non endogenous homoquinolinic acid discriminate between NMDAR2 receptor subunits. Neurochem. Int..

[96] DiFiglia M. (1990). Excitotoxic injury of the neostriatum: A model for Huntington’s disease. Trends Neurosci..

[97] Zádori D., Klivényi P., Vámos E., Fülöp F., Toldi J., Vécsei L. (2009). Kynurenines in chronic neurodegenerative disorders: Future therapeutic strategies. J. Neural Transm..

[98] Campesan S., Green E.W., Breda C., Sathyasaikumar K.V., Muchowski P.J., Schwarcz R., Kyriacou C.P., Giorgini F. (2011). The kynurenine pathway modulates neurodegeneration in a Drosophila model of Huntington’s disease. Curr. Biol..

[99] Mazarei G., Leavitt B.R. (2015). Indoleamine 2,3 Dioxygenase as a Potential Therapeutic Target in Huntington’s Disease. J. Huntington’s Dis..

[100] Zádori D., Nyiri G., Szonyi A., Szatmári I., Fülöp F., Toldi J., Freund T.F., Vécsei L., Klivényi P. (2011). Neuroprotective effects of a novel kynurenic acid analogue in a transgenic mouse model of Huntington’s disease. J. Neural Transm..

[101] Gellért L., Varga D., Ruszka M., Toldi J., Farkas T., Szatmári I., Fülöp F., Vécsei L., Kis Z. (2012). Behavioural studies with a newly developed neuroprotective KYNA-amide. J. Neural Transm..

[102] Varga D., Herédi J., Kanvasi Z., Ruszka M., Kis Z., Ono E., Iwamori N., Iwamori T., Takakuwa H., Vécsei L. (2015). Systemic L-kynurenine sulfate administration disrupts object recognition memory, alters open field behavior and decreases c-Fos immunopositivity in C57Bl/6 mice. Front. Behav. Neurosci..

[103] Stoy N., Mackay G.M., Forrest C.M., Christofides J., Egerton M., Stone T.W., Darlington L.G. (2005). Tryptophan metabolism and oxidative stress in patients with Huntington’s disease. J. Neurochem..

[104] Beal M.F., Matson W.R., Swartz K.J., Gamache P.H., Bird E.D. (1990). Kynurenine pathway measurements in Huntington’s disease striatum: Evidence for reduced formation of kynurenic acid. J. Neurochem..

[105] Jauch D., Urbanska E.M., Guidetti P., Bird E.D., Vonsattel J.P., Whetsell W.J., Schwarcz R. (1995). Dysfunction of brain kynurenic acid metabolism in Huntington’s disease: Focus on kynurenine aminotransferases. J. Neurol. Sci..

[106] Heyes M.P., Jordan E.K., Lee K., Saito K., Frank J.A., Snoy P.J., Markey S.P., Gravell M. (1992). Relationship of neurologic status in macaques infected with the simian immunodeficiency virus to cerebrospinal fluid quinolinic acid and kynurenic acid. Brain Res..

[107] Beal M.F., Matson W.R., Storey E., Milbury P., Ryan E.A., Ogawa T., Bird E.D. (1992). Kynurenic acid concentrations are reduced in Huntington’s disease cerebral cortex. J. Neurol. Sci..

[108] Guidetti P., Bates G.P., Graham R.K., Hayden M.R., Leavitt B.R., MacDonald M.E., Slow E.J., Wheeler V.C., Woodman B., Schwarcz R. (2006). Elevated brain 3-hydroxykynurenine and quinolinate levels in Huntington disease mice. Neurobiol. Dis..

[109] Zwilling D., Huang S.Y., Sathyasaikumar K.V., Notarangelo F.M., Guidetti P., Wu H.Q., Lee J., Truong J., Andrews-Zwilling Y., Hsieh E.W. (2011). Kynurenine 3-monooxygenase inhibition in blood ameliorates neurodegeneration. Cell.

[110] Beal M.F., Vécsei L., Vécsei L., Freese A., Swartz K.J., Beal M.F. (1992). Excitatory amino acids in the pathogenesis of neurodegenerative disorders. Neurological Disorders: Novel Experimental and Therapeutic Strategies.

[111] Sas K., Robotka H., Toldi J., Vécsei L. (2007). Mitochondria, metabolic disturbances, oxidative stress and the kynurenine system, with focus on neurodegenerative disorders. J. Neurol. Sci..

[112] Chen Y., Stankovic R., Cullen K.M., Meininger V., Garner B., Coggan S., Grant R., Brew B.J., Guillemin G.J. (2010). The kynurenine pathway and inflammation in amyotrophic lateral sclerosis. Neurotox. Res..

[113] Ilzecka J., Kocki T., Stelmasiak Z., Turski W.A. (2003). Endogenous protectant kynurenic acid in amyotrophic lateral sclerosis. Acta Neurol. Scand..

[114] Zoccolella S., Beghi E., Palagano G., Fraddosio A., Guerra V., Samarelli V., Lepore V., Simone I.L., Lamberti P., Serlenga L. (2007). Riluzole and amyotrophic lateral sclerosis survival: A population-based study in southern Italy. Eur. J. Neurol..

[115] Jaiswal M.K. (2019). Riluzole and edaravone: A tale of two amyotrophic lateral sclerosis drugs. Med. Res. Rev..

[116] Chen Y., Meininger V., Guillemin G.J. (2009). Recent advances in the treatment of amyotrophic lateral sclerosis. Emphasis on kynurenine pathway inhibitors. Cent. Nerv. Syst. Agents Med. Chem..

[117] Raine C.S., Scheinberg L.C. (1988). On the immunopathology of plaque development and repair in multiple sclerosis. J. Neuroimmunol..

[118] Kwidzinski E., Bunse J., Aktas O., Richter D., Mutlu L., Zipp F., Nitsch R., Bechmann I. (2005). Indolamine 2,3-dioxygenase is expressed in the CNS and down-regulates autoimmune inflammation. FASEB J..

[119] Chiarugi A., Cozzi A., Ballerini C., Massacesi L., Moroni F. (2001). Kynurenine 3-mono oxygenase activity and neurotoxic kynurenine metabolites increase in the spinal cord of rats with experimental allergic encephalomyelitis. Neuroscience.

[120] Hartai Z., Klivényi P., Janáky T., Penke B., Dux L., Vécsei L. (2005). Kynurenine metabolism in multiple sclerosis. Acta Neurol. Scand..

[121] Rudzite V., Berzinsh J., Grivane I., Fuchs D., Baier-Bitterlich G., Wachter H. (1996). Serum tryptophan, kynurenine, and neopterin in patients with Guillain-Barresyndrome (GBS) and multiple sclerosis (MS). Adv. Exp. Med. Biol..

[122] Jovanovic F., Candido K.D., Knezevic N.N. (2020). The Role of the Kynurenine Signaling Pathway in Different Chronic Pain Conditions and Potential Use of Therapeutic Agents. Int. J. Mol. Sci..

[123] Rejdak K., Petzold A., Kocki T., Kurzepa J., Grieb P., Turski W.A., Stelmasiak Z. (2007). Astrocytic activation in relation to inflammatory markers during clinical exacerbation of relapsing-remitting multiple sclerosis. J. Neural Transm..

[124] Rejdak K., Bartosik-Psujek H., Dobosz B., Kocki T., Grieb P., Giovannoni G., Turski W.A., Stelmasiak Z. (2002). Decreased level of kynurenic acid in cerebrospinal fluid of relapsing-onset multiple sclerosis patients. Neurosci. Lett..

[125] Lim C.K., Bilgin A., Lovejoy D.B., Tan V., Bustamante S., Taylor B.V., Bessede A., Brew B.J., Guillemin G.J. (2017). Kynurenine pathway metabolomics predicts and provides mechanistic insight into multiple sclerosis progression. Sci. Rep..

[126] Aeinehband S., Brenner P., Stahl S., Bhat M., Fidock M.D., Khademi M., Olsson T., Engberg G., Jokinen J., Erhardt S. (2016). Cerebrospinal fluid kynurenines in multiple sclerosis; relation to disease course and neurocognitive symptoms. Brain Behav. Immun..

[127] Jönsson S., Andersson G., Fex T., Fristedt T., Hedlund G., Jansson K., Abramo L., Fritzson I., Pekarski O., Runström A. (2004). Synthesis and biological evaluation of new 1,2-dihydro-4-hydroxy-2-oxo-3-quinolinecarboxamides for treatment of autoimmune disorders: Structure-activity relationship. J. Med. Chem..

[128] Majláth Z., Annus A., Vécsei L. (2018). Kynurenine system and multiple sclerosis, pathomechanism and drug targets with an emphasis on laquinimod. Curr. Drug Targ..

[129] Nedelcu J., Reinbach C., Riedler P., Brendel M., Rominger A., Kaye J., Behrangi N., Jiangshan Z., Schmitz C., Ki M. (2020). Laquinimod ameliorates secondary brain inflammation. Neurobiol. Dis..

[130] Comi G., Pulizzi A., Rovaris M., Abramsky O., Arbizu T., Boiko A., Gold R., Havrdova E., Komoly S., Selmaj K.W. (2008). Effect of laquinimod on MRI-monitored disease activity in patients with relapsing-remitting multiple sclerosis: A multicentre, randomised, double-blind, placebo-controlled phase IIb study. Lancet.

[131] Williams W.L., Hoff-Jorgensen E., Snell E.E. (1949). Determination and properties of an unidentified growth factor required by Lactobacillus bulgaricus. J. Biol. Chem..

[132] Snell E.E., Brown G.M. (1953). Pantethine and related forms of the Lactobacillus bulgaricus factor (LBF). Adv. Enzymol. Relat. Areas Mol. Biol..

[133] Ono S., Kameda K., Abiko Y. (1974). Metabolism of panthethine in the rat. J. Nutr. Sci. Vitaminol..

[134] Reichlin S., Bollinger-Gruber J.A. (1985). Pantethine, a cysteamine precursor, depletes immunoreactive somatostatin and prolactin in the rat. Endocrinology.

[135] Jeitner T.M., Oliver J.R. (1990). The depletion of plasma prolactin by pantethine in oestrogen primed hyperprolactinaemic rats. J. Endocrinol..

[136] Vécsei L., Widerlov E., Alling C. (1989). Effects of pantethine, cysteamine and pantothenic acid on open-field behavior and brain catecholamines in rats. Arch. Int. Pharmacodyn. Ther..

[137] Nagiel-Ostaszewski I., Lau-Cam C.A. (1990). Protection by pantethine, pantothenic acid and cystamine against carbontetrachloride-induced hepatotoxicity in the rat. Res. Commun. Chem. Pathol. Pharmacol..

[138] van Gijsel-Bonnello M., Baranger K., Benech P., Rivera S., Khrestchatisky M., de Reggi M., Gharib B. (2017). Metabolic changes and inflammationin cultured astrocytes from the 5×FAD mouse model of Alzheimer’s disease: Alleviation by pantethine. PLoS ONE.

[139] Baranger K., van Gijsel-Bonnello M., Stephan D., Carpentier W., Rivera S., Khrestchatisky M., Gharib B., De Reggi M., Benech P. (2019). Long-term pantethine treatment counteracts pathologic gene dysregulation and decreases Alzheimer’s disease pathogenesis in a transgenic mouse model. Neurotherapeutics.

[140] Schapira A.H., Cooper J.M., Dexter D., Jenner P., Clark J.B., Marsden C.D. (1989). Mitochondrial complex I deficiency in Parkinson’s disease. Lancet.

[141] Cornille E., Abou-Hamdan M., Khrestchatisky M., Nieoullon A., de Reggi M., Gharib B. (2010). Enhancement of L-3-hydroxybutyryl-CoA dehydrogenase activity and circulating ketone body levels by pantethine. Relevance to dopaminergic injury. BMC Neurosci..

[142] Abou-Hamdan M., Cornille E., Khrestchatisky M., de Reggi M., Gharib B., Rana A.Q. (2011). The Energy Crisis in Parkinson’s Disease: A Therapeutic Target. Etiology and Pathophysiology of Parkinson’s Disease.

[143] Imamura K., Takeshima T., Kashiwaya Y., Nakaso K., Nakashima K. (2006). D-betahydroxybutyrate protects dopaminergic SH-SY5Y cells in a rotenone model of Parkinson’s disease. J. Neurosci. Res..

[144] Zhao Q., Stafstrom C.E., Fu D.D., Hu Y., Holmes G.L. (2004). Detrimental effects of the ketogenic diet on cognitive function in rats. Pediatric Res..

[145] Kessler R.C., Berglund P., Demler O., Jin R., Koretz D., Merikangas K.R., Rush J., Walters E.E., Wang P.S. (2003). The epidemiology of major depressive disorder: Results from the National Comorbidity Survey Replication (NCS-R). JAMA.

[146] Sackeim H.A. (2001). The definition and meaning of treatment-resistant depression. J. Clin. Psychiatry.

[147] Slattery D.A., Hudson A.L., Nutt D.J. (2004). Invited review: The evolution of depressant mechanisms. Fundam. Clin. Pharmacol..

[148] Duman R.S. (2004). Role of neurotrophic factors in the etiology and treatment of mood disorders. Neuromolecular Med..

[149] Borrell-Pages M., Canals J.M., Cordelieres F.P., Parker J.A., Pineda J.R., Grange G., Bryson E.A., Guillermier M., Hirsch E., Hantraye P. (2006). Cystamine and cysteamine increase brain levels of BDNF in Huntington disease via HSJ1b and transglutaminase. J. Clin. Invest..

[150] Corden B.J., Schulman J.D., Schneider J.A., Thoene J.G. (1981). Adverse reactions to oral cysteamine use in nephropathic cystinosis. Dev. Pharmacol. Ther..

[151] Avner E.D., Ellis D., Jaffe R. (1983). Veno-occlusive disease of the liver associated with cysteamine treatment of nephropathic cystinosis. J. Pediatrics.

[152] Vécsei L., Widerlov E. (1990). Preclinical and clinical studies with cysteamine and pantethine related to the central nervous system. Prog. Neuro-Psychopharmacol. Biol. Psychiatry.

[153] Drevets W.C. (1994). Geriatric depression: Brain imaging correlates and pharmacologic considerations. J. Clin. Psychiatry.

[154] Kruer M.C., Boddaert N., Schneider S.A., Houlden H., Bhatia K.P., Gregory A., Anderson J.C., Rooney W.D., Hogarth P., Hayflick S.J. (2012). Neuroimaging features of neurodegeneration with brain ironaccumulation. Am. J. Neuroradiol..

[155] Brunetti D., Dusi S., Giordano C., Lamperti C., Morbin M., Fugnanesi V., Marchet S., Fagiolari G., Sibon O., Moggio M. (2014). Pantethine treatment is effective in recovering the disease phenotype induced by ketogenic diet in a pantothenate kinase-associated neurodegeneration mouse model. Brain.

[156] Hayflick S.J. (2003). Pantothenate kinase-associated neurodegeneration (formerly Hallervorden-Spatz syndrome). J. Neurol. Sci..

[157] Gregory A., Westaway S.K., Holm I.E., Kotzbauer P.T., Hogarth P., Sonek S., Coryell J.C., Nguyen T.M., Nardocci N., Zorzi G. (2008). Neurodegeneration associated with genetic defects in phospho-lipase A(2). Neurology.

[158] Leonardi R., Rock C.O., Jackowski S., Zhang Y.M. (2007). Activation of human mitochondrial pantothenate kinase 2 by palmitoylcarnitine. Proc. Natl. Acad. Sci. USA.

[159] Rana A., Seinen E., Siudeja K., Muntendam R., Srinivasan B., van der Want J.J., Hayflick S., Reijngoud D.R., Kayser O., Sibon O.C.M. (2010). Pantethine rescues a Drosophila model for pantothenate kinase-associated neurodegeneration. Proc. Natl. Acad. Sci. USA.

[160] Zizioli D., Tiso N., Guglielmi A., Saraceno C., Busolin G., Giuliani R., Khatri D., Monti E., Borsani G., Argenton F. (2016). Knock-down of pantothenate kinase 2 severely affects the development of the nervous and vascular system in zebrafish, providing new insights into PKAN disease. Neurobiol. Dis..

[161] Lieber C.S. (1980). Alcohol, protein metabolism and liver injury. Gastroenterology.

[162] Myer R.D. (1978). Tetrahydroisoquinolines in the brain: The basis of an animal model of addiction. Alcohol. Clin. Exp. Res..

[163] Watanabe A., Hobara N., Kobayashi M., Nakatsukasa H., Nagashima H. (1985). Lowering of blood acetaldehyde but not ethanol concentrations by pantethine following alcohol ingestion: Different effects in flushing and nonflushing subjects. Alcohol. Clin. Exp. Res..

[164] Smith C.M., Israel B.C., Iannucci J., Marino K. (1987). Possible role of Acetyl-CoA in the inhibition of CoA biosynthesis by ethanol in rats. J. Nutr..

[165] Rivera-Calimlim L., Hartley D., Osterhout D. (1988). Effects of ethanol and pantothenic acid on brain acetylcholine synthesis. Br. J. Pharmacol..

[166] Branca D., Scutari G., Siliprandi N. (1984). Pantethine and pantothenate effect on the CoA content of rat liver. Int. J. Vitam. Nutr. Res..

[167] Bon G.B., Cazzolato G., Zago S., Avogaro P. (1985). Effects of pantethine on in-vitro peroxidation of low density lipoproteins. Atherosclerosis.

[168] Arsenio L., Bodria P., Magnati G., Strata A., Trovato R. (1986). Effectiveness of long-term treatment with pantethine in patients with dyslipidemia. Clin. Ther..

[169] Bertolini S., Donati C., Elicio N., Daga A., Cuzzolaro S., Marcenaro A., Saturnino M., Balestreri R. (1986). Lipoprotein changes induced by pantethine in hyperlipoproteinemic patients: Adults and children. Int. J. Clin. Pharmacol. Ther. Toxicol..

[170] McRae M.P. (2005). Treatment of hyperlipoproteinemia with pantethine: A review and analysis of efficacy and tolerability. Nutr. Res..

[171] Elmonem M.A., Veys K.R., Soliman N.A., van Dyck M., van den Heuvel L.P., Levtchenko E. (2016). Cystinosis: A review. Orphanet J. Rare Dis..

[172] Butler J.D., Zatz M. (1984). Pantethine and cystamine deplete cystine from cystinotic fibroblasts via efflux of cysteamine-cystine mixed disulfide. J. Clin. Invest..

[173] Clark J.I., Livesey J.C., Steele J.E. (1996). Delay or inhibition of rat lens opacification using pantethine and WR-77913. Exp. Eye Res..

[174] Nebbioso M., Pranno F., Pescosolido N. (2013). Lipoic acid in animal models and clinical use in diabetic retinopathy. Expert Opin. Pharmacother..

[175] Reed L.J. (1953). Metabolic functions of thiamine and lipoic acid. Physiol. Rev..

[176] Packer L., Witt E.H., Tritschler H.J. (1995). Alpha-Lipoic acid as a biological antioxidant. Free Radic. Biol. Med..

[177] Smith A.R., Shenvi S.V., Widlansky M., Suh J.H., Hagen T.M. (2004). Lipoic acid as a potential therapy for chronic diseases associated with oxidative stress. Curr. Med. Chem..

[178] Biewenga G.P., Haenen G.R., Bast A. (1997). The pharmacology of the antioxidant lipoic acid. Gen. Pharmacol. Vasc. Syst..

[179] Packer L., Tritschler H.J., Wessel K. (1997). Neuroprotection by the metabolic antioxidant alphalipoic acid. Free Radic. Biol. Med..

[180] Moini H., Packer L., Saris N.-E.L. (2002). Antioxidant and Prooxidant Activities of α-Lipoic Acid and Dihydrolipoic Acid. Toxicol. Appl. Pharmacol..

[181] Kagan V.E., Shvedova A., Serbinova E., Khan S., Swanson C., Powell R., Packer L. (1992). Dihydrolipoic acid-a universal antioxidant both in the membrane and in the aqueous phase. Biochem. Pharmacol..

[182] Moura F.A., de Andrade K.Q., dos Santos J.C., Goulart M.O. (2015). Lipoic Acid: Its Antioxidant and Anti-Inflammatory Role and Clinical Applications. Curr. Top. Med. Chem..

[183] Yan W., Li N., Hu X., Huang Y., Zhang W., Wang Q., Wang F., Wang C., Zhai X., Xu R. (2013). Effect of oral ALA supplementation on oxidative stress and insulin sensitivity among overweight/obese adults: A double-blinded, randomized, controlled, cross-over intervention trial. Int. J. Cardiol..

[184] Kim M.-S., Park J.-Y., Namkoong C., Jang P.-G., Ryu J.-W., Song H.-S., Yun J.-Y., Namgoong I.-S., Ha J., Park I.-S. (2004). Anti-obesity effects of alpha-lipoic acid mediated by suppression of hypothalamic AMP-activated protein kinase. Nat. Med..

[185] Thakurta I.G., Chattopadhyay M., Ghosh A., Chakrabarti S. (2012). Dietary supplementation with N-acetyl cysteine, α-tocopherol and α-lipoic acid reduces the extent of oxidative stress and proinflammatory state in aged rat brain. Biogerontology.

[186] Al Abdan M. (2012). Alfa-lipoic acid controls tumor growth and modulates hepatic redox state in Ehrlich-ascites-carcinoma-bearing mice. Sci. World J..

[187] Martins V.D., Manfredini V., Peralba M.C.R., Benfato M.S. (2009). Alpha-lipoic acid modifies oxidative stress parameters in sickle cell trait subjects and sickle cell patients. Clin. Nutr..

[188] Bae S.C., Jung W.J., Lee E.J., Yu R., Sung M.K. (2009). Effects of antioxidant supplements intervention on the level of plasma inflammatory molecules and disease severity of rheumatoid arthritis patients. J. Am. Coll. Nutr..

[189] Mauro G.L., Cataldo P., Barbera G., Sanfilippo A. (2014). α-Lipoic acid and superoxide dismutase in the management of chronic neck pain: A prospective randomized study. Drugs R D.

[190] Gu X., Zhang S., Wu J., Tang Z., Lu Z., Li H., Liu C., Chen L., Ning G. (2010). Efficacy and safety of high-dose α-lipoic acid in the treatment of diabetic polyneuropathy. Zhonghua Yi Xue Za Zhi.

[191] Ali A.M., Awad T.G., Al-Adl N.M. (2010). Efficacy of combined topiramate/thioctic acid therapy in migraine prophylaxis. Saudi Pharm. J..

[192] Trivedi P.P., Jena G.B. (2013). Role of α-lipoic acid in dextran sulfate sodium-induced ulcerative colitis in mice: Studies on inflammation, oxidative stress, DNA damage and fibrosis. Food Chem. Toxicol..

[193] Shay K.P., Moreau R.F., Smith E.J., Smith A.R., Hagen T.M. (2009). Alpha-lipoic acid as a dietary supplement: Molecular mechanisms and therapeutic potential. Biochim. Biophys. Acta.

[194] Zhang W.J., Frei B. (2001). Alpha-lipoic acid inhibits TNF-alpha-induced NF-kappaB activation and adhesion molecule expression in human aortic endothelial cells. FASEB J..

[195] Ono K., Hirohata M., Yamada M. (2006). Alpha-lipoic acid exhibits anti-amyloidogenicity for betaamyloid fibrils in vitro. Biochem. Biophys. Res. Commun..

[196] Hager K., Marahrens A., Kenklies M., Riederer P., Münch G. (2001). Alpha-lipoic acid as a new treatment option for Azheimer type dementia. Arch. Gerontol. Geriatr..

[197] Venigalla M., Gyengesi E., Sharman M.J., Münch G. (2016). Novel promising therapeutics against chronic neuroinflammation and neurodegeneration in Alzheimer’s disease. Neurochem. Int..

[198] Richardson J.R., Hossain M.M. (2013). Microglial ion channels as potential targets for neuroprotection in Parkinson’s disease. Neural Plast..

[199] Zhang Z., Hou L., Song J.-L., Song N., Sun Y.-J., Lin X., Wang X.-L., Zhang F.-Z., Ge Y.-L. (2014). Pro-inflammatory cytokine-mediated ferroportin down-regulation contributes to the nigral iron accumulation in lipopolysaccharide-induced Parkinsonian models. Neuroscience.

[200] Dutta G., Zhang P., Liu B. (2008). The lipopolysaccharide Parkinson’s disease animal model: Mechanistic studies and drug discovery. Fundam. Clin. Pharmacol..

[201] He Q., Yu W., Wu J., Chen C., Lou Z., Zhang Q., Zhao J., Wang J., Xiao B. (2013). Intranasal LPS-mediated Parkinson’s model challenges the pathogenesis of nasal cavity and environmental toxins. PLoS ONE.

[202] Zhang W.J., Wei H., Hagen T., Frei B. (2007). Alpha-lipoic acid attenuates LPS induced inflammatory responses by activating the phosphoinositide 3-kinase/Akt signaling pathway. Proc. Natl. Acad. Sci. USA.

[203] Tancheva L.P., Lazarova M.I., Alexandrova A.V., Dragomanova S.T., Nicoletti F., Tzvetanova E.R., Hodzhev Y.K., Kalfin R.E., Miteva S.A., Mazzon E. (2020). Neuroprotective mechanisms of three natural antioxidants on a rat model of Parkinson’s disease: A Comparative Study. Antioxidants.

[204] Beal M.F., Ferrante R.J. (2004). Experimental therapeutics in transgenic mouse models of Huntington’s disease. Nat. Rev. Neurosci..

[205] Brouillet E., Guyot M.C., Mittoux V., Altairac S., Conde F., Palfi S., Hantraye P. (1998). Partial inhibition of brain succinate dehydrogenase by 3-nitropropionic acid is sufficient to initiate striatal degeneration in rat. J. Neurochem..

[206] Mehrotra A., Sood A., Sandhir R. (2015). Mitochondrial modulators improve lipid composition and attenuate memory deficits in experimental model of Huntington’s disease. Mol. Cell. Biochem..

[207] Andreassen O.A., Ferrante R.J., Dedeoglu A., Beal M.F. (2001). Lipoic acid improves survival in transgenic mouse models of Huntington’s disease. NeuroReport.

[208] Franklin R.J., Ffrench-Constant C., Edgar J.M., Smith K.J. (2012). Neuroprotection and repair in multiple sclerosis. Nat. Rev. Neurol..

[209] Smith K.J., Kapoor R., Felts P.A. (1999). Demyelination: The role of reactive oxygen and nitrogen species. Brain Pathol..

[210] Tanaka M., Vécsei L. (2020). Monitoring the Redox Status in Multiple Sclerosis. Biomedicines.

[211] Morini M., Roccatagliata L., Dell’Eva R., Pedemonte E., Furlanc R., Minghelli S., Giunti D., Pfeffer U., Marchese M., Noonan D. (2004). Alpha-lipoic acid is effective in prevention and treatment of experimental autoimmune encephalomyelitis. J. Neuroimmunol..

[212] Chaudhary P., Marracci G.H., Bourdette D.N. (2006). Lipoic acid inhibits expression of ICAM-1 and VCAM-1 by CNS endothelial cells and T cell migration into the spinal cord in experimental autoimmune encephalomyelitis. J. Neuroimmunol..

[213] Chaudhary P., Marracci G., Yu X., Galipeau D., Morris B., Bourdette D. (2011). Lipoic acid decreases inflammation and confers neuroprotection in experimental autoimmune optic neuritis. J. Neuroimmunol..

[214] Marracci G.H., Jones R.E., McKeon G.P., Bourdette D.N. (2002). Alpha lipoic acid inhibits T cell migration into the spinal cord and suppresses and treats experimental autoimmune encephalomyelitis. J. Neuroimmunol..

[215] Jones R.E., Moes N., Zwickey H., Cunningham C.L., Gregory W.L., Oken B. (2008). Treatment of experimental autoimmune encephalomyelitis with alpha lipoic acid and associative conditioning. Brain Behav. Immun..

[216] Wang K.-C., Tsai C.-P., Lee C.-L., Chen S.-Y., Lin G.-J., Yen M.-H., Sytwu H.-K., Chen S.-J. (2013). Alpha-Lipoic acid enhances endogenous peroxisome-proliferator-activated receptor-gamma to ameliorate experimental autoimmune encephalomyelitis in mice. Clin. Sci. (Lond.).

[217] Yadav V., Marracci G., Lovera J., Woodward W., Bogardus K., Marquardt W., Shinto L., Morris C., Bourdette D. (2005). Lipoic acid in multiple sclerosis: A pilot study. Mult. Scler..

[218] Khalili M., Azimi A., Izadi V., Eghtesadi S., Mirshafiey A., Sahraian M.A., Motevalian A., Norouzi A., Sanoobar M., Eskandari G. (2014). Does lipoic acid consumption affect the cytokine profile in multiple sclerosis patients: A double-blind, placebo-controlled, randomized clinical trial. Neuroimmunomodulation.

